# Divergent COVID-19 Disease Trajectories Predicted by a DAMP-Centered Immune Network Model

**DOI:** 10.3389/fimmu.2021.754127

**Published:** 2021-10-28

**Authors:** Judy D. Day, Soojin Park, Benjamin L. Ranard, Harinder Singh, Carson C. Chow, Yoram Vodovotz

**Affiliations:** ^1^ Department of Mathematics, University of Tennessee, Knoxville, TN, United States; ^2^ Department of Electrical Engineering & Computer Science, University of Tennessee, Knoxville, TN, United States; ^3^ Department of Neurology & Division of Critical Care and Hospital Neurology, Columbia University College of Physicians and Surgeons, New York Presbyterian Hospital – Columbia University Irving Medical Center, New York, NY, United States; ^4^ Program for Hospital and Intensive Care Informatics, Department of Neurology, Columbia University College of Physicians and Surgeons, New York, NY, United States; ^5^ Division of Pulmonary, Allergy & Critical Care Medicine, Department of Medicine, Columbia University College of Physicians and Surgeons, New York Presbyterian Hospital – Columbia University Irving Medical Center, New York, NY, United States; ^6^ Department of Immunology, University of Pittsburgh, Pittsburgh, PA, United States; ^7^ Center for Systems Immunology, University of Pittsburgh, Pittsburgh, PA, United States; ^8^ Mathematical Biology Section, Laboratory of Biological Modeling, National Institute of Diabetes and Digestive and Kidney Diseases, Bethesda, MD, United States; ^9^ Department of Surgery, University of Pittsburgh, Pittsburgh, PA, United States; ^10^ Center for Inflammation and Regeneration Modeling, McGowan Institute for Regenerative Medicine, University of Pittsburgh, Pittsburgh, PA, United States

**Keywords:** COVID-19, SARS-CoV2, mathematical model, immune response, inflammation, DAMP, danger signal

## Abstract

COVID-19 presentations range from mild to moderate through severe disease but also manifest with persistent illness or viral recrudescence. We hypothesized that the spectrum of COVID-19 disease manifestations was a consequence of SARS-CoV-2-mediated delay in the pathogen-associated molecular pattern (PAMP) response, including dampened type I interferon signaling, thereby shifting the balance of the immune response to be dominated by damage-associated molecular pattern (DAMP) signaling. To test the hypothesis, we constructed a parsimonious mechanistic mathematical model. After calibration of the model for initial viral load and then by varying a few key parameters, we show that the core model generates four distinct viral load, immune response and associated disease trajectories termed “patient archetypes”, whose temporal dynamics are reflected in clinical data from hospitalized COVID-19 patients. The model also accounts for responses to corticosteroid therapy and predicts that vaccine-induced neutralizing antibodies and cellular memory will be protective, including from severe COVID-19 disease. This generalizable modeling framework could be used to analyze protective and pathogenic immune responses to diverse viral infections.

## Introduction

The COVID-19 pandemic has been characterized by diverse clinical manifestations that have been associated with varying host immuno-inflammatory responses to the severe acute respiratory syndrome coronavirus 2 (SARS-CoV-2) virus. These phenotypes, which include large numbers of asymptomatic carriers (1–3), have ranged from moderate to severely ill patients (4, 5), those with extra-pulmonary manifestations (6), and with persistent disease [“long-haulers” (7–9)]. Patients that have underlying co-morbidities associated with chronic inflammation, such as obesity (10–12), diabetes (12–14), and cardiovascular disease (13, 14), are especially prone to severe disease presentations. An understanding of why such varied pathophysiologic manifestations occur, and the development of patient-specific treatment modalities, is of utmost importance.

In the early stages of the pandemic, attempts had been made to predict the host immune and inflammatory responses to SARS-CoV-2 in the context of the known biology of other coronaviruses (15). More recently, numerous studies have used approaches drawn from systems immunology to perform high-dimensional profiling of the immune and inflammatory responses in COVID-19 patients. These include machine learning analyses aimed at defining features that may aid diagnosis or therapy. The studies have emphasized the presence of an imbalanced or overwhelming immune-inflammatory response in severe disease (16–18).

SARS-CoV-2 likely generates diverse immune and pathophysiologic responses because the adaptive immune system is naïve to it unless there is some cross-immunity due to prior infection with other coronaviruses (19). To clear a viral infection, adaptive immunity must be activated, and this happens in one of two ways. The first is through immune memory in the form of memory T or B cells. If memory does not exist [or cross reactivity with other beta coronaviruses is weak (19)], as will generally be the case with SARS-CoV-2, the adaptive immune system must be alerted *de novo* through the activation of innate immunity; however, recent evidence suggests that this pathway [specifically the production of type I interferons (20, 21)] is diminished in COVID-19 patients (15, 18, 22–26).

We hypothesized that SARS-CoV-2 attenuation of pathogen-associated molecular patterns (PAMPs)-dependent innate immune responses exposes an alternate pathway for activation of innate and adaptive immunity which is instigated by cellular damage. This results in a delay of PAMP-activated innate immunity as the virus first causes cellular or tissue dysfunction that induces a nonspecific inflammatory response *via* the release of damage-associated molecular pattern molecules (DAMPs) (27–29). The DAMP-mediated inflammatory response primes the adaptive immune response through the activation of dendritic cells and other antigen-presenting cells, resulting in the release of various pro-inflammatory cytokines (30, 31). Notably, such an inflammatory response can itself cause additional damage and dysfunction (27); furthermore, the inflammatory and adaptive immune components can inhibit each other (32, 33). Within such an overall framework, the relative timing and amplitude of these complex dynamical interactions could be impacted by genetic variation as well as by co-morbidities, thereby resulting in the diverse manifestations of COVID-19 pathophysiology that have been observed clinically.

To test the core hypothesis as the underlying basis of diverse manifestations of SARS-CoV-2 infection and COVID-19 disease, we formulated a parsimonious, mechanistic mathematical model of viral infection and the ensuing systemic inflammation as well as innate and adaptive immune cell responses. In accordance with our hypothesis our model partitioned innate immune cell activation *via* either PAMPs or DAMPs, the latter emanating from virus-infected host cells, both of which can lead to the induction of an adaptive immune response to viral infection. Although robust PAMP signaling generally occurs during viral infections (20, 21), as noted above recent studies suggest that this can be attenuated significantly in COVID-19 patients, resulting in impaired type I interferon production (15, 18, 22–26). Thus, our model was designed and tuned for the context in which virus infection leads initially to damage/dysfunction and promotes innate immune responses and systemic inflammation. Alterations of few key parameters within the model generated four distinct dynamic patterns of viral load, immune response and associated disease manifestations. Importantly, these prototypical infectious disease patterns termed “archetypes” were qualitatively reflected in clinical data from hospitalized COVID-19 patients. Simulations with our model also reproduced key features of anti-inflammatory treatment and predicted protective responses induced by vaccination.

## Materials and Methods

The overall design and execution of this study involved 1) the generation of a parsimonious mathematical model based on our core hypothesis [namely that a DAMP-centered response to SARS-CoV-2 can result in dynamically distinct biological and clinical trajectories (archetypal COVID-19 responses or “COVID-19 archetypes”)] depending on the relative interactions among virus-infected cells, the innate immune response to DAMPs that are released from damaged or dysfunctional tissue, and the adaptive immune response triggered by innate immune activation); 2) initial calibration of viral inoculum using published data on experimental SARS-CoV-2 infection; 3) further calibration of viral inoculum to data from COVID-19 patients followed by model simulations revealing distinct disease trajectories that correspond with clinical data from exemplary patients; and finally 4) simulations of anti-inflammatory therapy and COVID-19 vaccination.

### Mathematical Model of the Immune-Inflammatory Response to SARS-CoV-2

The model used in all simulations obeyed the following differential equations:


(1) $$\frac{{dC}_{V}}{dt}=k_{vg}C_{V}\left(1-k_{vs}C_{V}\right)f_{1}\left(1,x_{vainv}A\right)-k_{va}AC_{V}$$

(2) $$\frac{dD}{dt}=k_{dv}f_{2}\left(C_{V},x_{dv}\right)+k_{di}f_{2}\left(I,x_{di}\right)-\mu_{d}D$$

(3) $$\frac{dI}{dt}=s_{ir}\frac{R}{\mu_{ir}+R}-\mu_{i}I$$

(4) $$dAdt=k_{ai}\frac{s_{a0}I}{\mu_{a0}+k_{ai}I}\frac{x_{1ai}^{2}}{x_{1ai}^{2}+I^{2}}+k_{ag}C_{V}\frac{A^{2}}{x_{ag}^{2}+A^{2}}-\mu_{a}+A_{e}$$

where


(5) $$R=\left(k_{id}D+k_{ii}I\right)f_{1}\left(1,x_{iainv}A\right)=\frac{k_{id}D+k_{ii}I}{1+{\left(x_{iainv}A\right)}^{2}};$$

and


$$f_{1}(x,y)=\frac{x}{1+y^{2}}\text{ for vaccination simulations but }f_{1}\left(x,y\right)=1\text{ otherwise};\text{ and }f_{2}\left(x,y\right)=\frac{x^{6}}{y^{6}+x^{6}}.$$

*C_V_*, is scaled such that a value of C_V_ = 10^−6^ corresponds to one infected cell. *I, D*, and *A* have arbitrary units. Parameters are described in **Table S2**. Most of the values were taken from previous models (34, 35) while others were estimated for the present application. The viral dynamics were adapted from an influenza model (36). The dynamical attractors were robust to wide variations in the parameter values. The differences between archetypes were due to changes in only four parameters (*k_di_*; *x_di_*; *x_iainv_*; and *k_ai_*), while the others were fixed. The detailed derivation and motivation for the model are in the **Supplementary Materials**.

Given the abstract nature of the compartments, no attempt was made to match the model quantitatively to the clinical scenarios except for the initial value of *C_V._* In SARS-CoV-2 infection of rhesus macaque monkeys (37), the viral peak occurred around day 5 after an initial intratracheal challenge with SARS-CoV-2 at 1x10^6^ 50% tissue-culture infectious doses (TCID_50_). At a starting *C_V_* value of 1x10^6^, our model predicted a peak time for the viral infection at 8 days. An exploration of the impact of increasing initial viral load on the time to peak infection for each COVID-19 archetype is detailed in **Table S2**. Based on this initial calibration, we calculated the typical initial *C_V_* value for human COVID-19 patients. This is difficult to do based on previously published data [e.g (17)], since those studies are based on patients that were already infected at the time of hospitalization and since data were obtained at only a few time points. We reasoned that granular data on patients hospitalized for reasons other than COVID-19, either with recent negative or initially negative screening tests, that became SARS-CoV-2-positive in the hospital setting (termed “incidental COVID-19”) would address these shortcomings. Accordingly, time of symptoms or PCR positivity data were obtained from “incidental COVID-19” patients hospitalized at New York Presbyterian Hospital (Columbia) between March 13 - August 5, 2020 (**Table 1**). SARS-CoV-2 infection was ascertained by RT-PCR tests in nasal swabs either as a screening test for procedures or triggered by symptoms including cough or fever. In this cohort, the time to virus positivity was estimated to be 11.3 ± 5.3 (n=8 incidental COVID-19 patients; range 4-22) days. From these data, we calibrated to a *C_V_* peak time of 11.1 ± 4.5 days (n=7 archetypal scenarios; range 8.5-19.1 days) in subsequent simulations based on an initial *C_V_* dose equivalent to 8 x 10^5^ virus-infected cells (**Table S2**), in general agreement with the aforementioned results in non-human primates.

**Table 1:** Incidental Patient data to calculate mean time to positive PCR test.

Age	Recent negative test prior to hospital admission	SARS-CoV-2 PCR at Admission			When Symptoms Started			Severe COVID-19	Days from negative test to Positive Test or to Symptoms
		Initial Test Positive	Initial Test Negative	Not tested	No COVID symptoms during admission	Mild symptoms present at admission	Develop symptoms while hospitalized		
40s	X	X			X				11
50s	X	X			X				22
70s	X	X				X			15
70s	X	X					X		11
70s			X				X		9
50s			X		X				8
50s				X	X				4
50s				X			X	X	10

Incidental patients were admitted for reasons other than COVID-19 and SARS-CoV-2 PCR tests were positive during hospitalization (either with symptoms or on a screening test). These eight had either prior recent negative SARS-CoV-2 PCR tests, were negative on admission, or were not tested on admission.

All computations were performed on MATLAB (version *9.7.0.1190202, R2019b*) and XPPAUT; and the model code is available (see **Auxiliary Supplementary Materials** for model code).

### Study Population

COVID-19 patient data were obtained from a prospective, observational COVID-19 cohort study that took place at New York-Presbyterian Hospital affiliated with Columbia University Irving Medical Center in northern Manhattan; the study was approved by the institutional review board at Columbia University Irving Medical Center (protocol AAAT0120) and requirement for informed consent was waived given the study design and ongoing pandemic. Adult patients (aged > 18 years) admitted between March 13, 2020 to August 5, 2020 who were diagnosed with laboratory-confirmed COVID-19 were identified through daily review of unit admission logs in the electronic medical record (n=657). Clinical, biomarker, and treatment data were collected. SARS-CoV-2 testing was performed using RT-PCR of nasopharyngeal swab samples and processed in the clinical microbiology laboratory of the New York-Presbyterian Hospital.

Not all patients from the COVID cohort study were included in this current study, as a qualitative comparison was intended rather than a statistical evaluation. Cohort patients were sorted for priority by the following criteria: (1) admission > 60 days or patients who died, (2) no treatment with steroids, and (3) most available values of SOFA scores, high sensitivity C-reactive protein values, creatinine values, or high sensitivity Troponin-T values; eight patients were included after comparison with simulation data. One of these patients had quite prolonged steroid administration (given for non-COVID-19 medical reasons) and was included for comparison with the uncontrolled infection simulation. Eight patients with incidental COVID-19 infections or recent negative SARS-CoV-2 PCR testing were identified in the cohort, and their interval times to symptoms or PCR positivity were summarized. In total, sixteen patients from the COVID cohort study were included in this qualitative comparison. Relevant data on this cohort are described in **Table 1** and **Auxiliary Supplementary Materials**.

### Diagnostics and Clinical Biomarkers

Patients were tested for SARS-CoV-2 using Roche Cobas 6800 (Roche Diagnostics, Indianapolis, IN, USA; FDA approved) or Cepheid Xpert Xpress (Sunnyvale, CA, USA; FDA approved), depending on testing platform availability. The nucleic acid amplification testing (NAAT) targeted E gene (SARS-specific) and SARS-CoV-2 specific N2 gene (Cepheid) or ORF gene (Roche). Cycle times, when available, are reported graphically as a visually intuitive index of (45-cycle time).

Laboratory results (from blood) are reported graphically including creatinine, high-sensitivity Troponin-T, high-sensitivity C-reactive protein, interleukin-6, D-dimer, and absolute monocyte count (automated). Creatinine is a biomarker of kidney function and is measured nearly daily (if not more as indicated) during hospitalization; reference ranges varied from 0.5-0.95 mg/dL to 0.7-1.30 mg/dL and are indicated accurately per patient. High-sensitivity Troponin-T is a biomarker of cardiac damage, and is measured as needed during hospitalization, in response to chest pain, electrocardiogram changes, or hemodynamic instability; reference ranges varied from < 14 ng/L to <22 ng/L and are indicated accurately per patient. C-reactive protein (CRP) is made by the liver and is released into the bloodstream in response to inflammation. High-sensitivity C-reactive protein was measured in the context of known COVID infection with varying frequency; reference range was 0-10 mg/L (high value 3.1-10 mg/L), with a maximum reported value of 300. Interleukin-6 assay (Cobas Elecys electrochemiluminescence immunoassay; FDA EUA) is elevated in the presence of an inflammatory response and was measured in the context of known COVID infection with varying frequency; reference range is less than or equal to 7 pg/mL, with a maximum reported value of 315. D-dimer (STAGO immune-turbidimetric assay) was measured in the context of known COVID infection with varying frequency; reference range is less than or equal to 0.8 microg/mL FEU. Absolute monocyte counts (automated) were often measured in routine hospitalized clinical care, as a differential of the white blood cells within the complete blood cell panel, this was examined as a surrogate of inflammation; the reference range varied from 0.1-0.9 x 10^3/^μL to 0.2-0.7 x 10^3^/μL to 0.22-1 x 10^3^/μL and are indicated accurately per patient.

## Results

### Model Construction: A DAMP-Centered Theoretical Model Predicts Four COVID-19 Infection and Disease Trajectories

**Figure 1:**
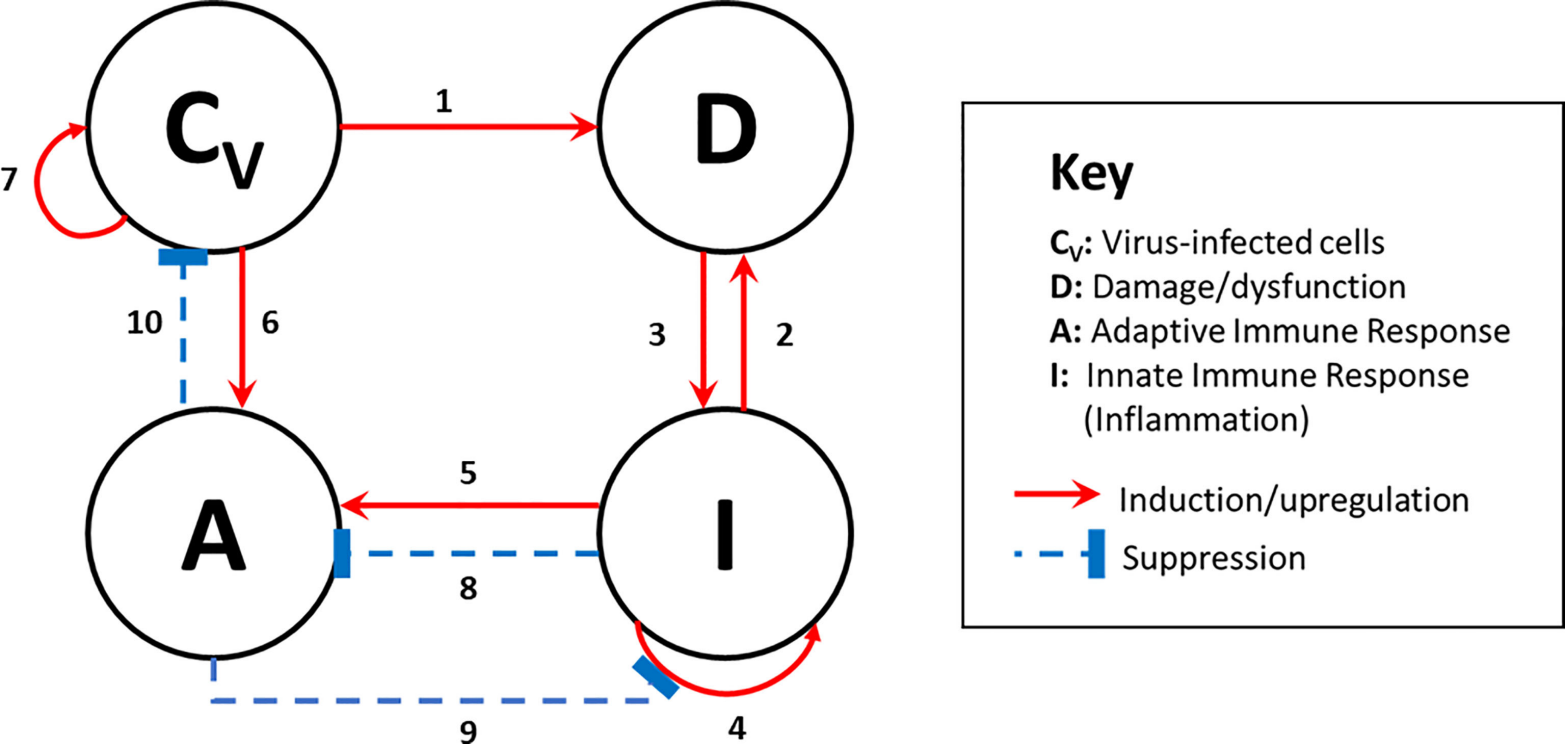
Model Interaction Diagram. Beginning at the top left of the diagram and explaining in a clockwise direction: The presence of virus-infected cells, *C_V_*, initiates the induction of damaged or dysfunctional tissue (arrow 1), modeled with the variable *D*. The lysis of virus-infected cells will lead to subsequent infection of other cells (arrow 7). The release of DAMPs from virus-infected cells occurs prior to the release of type I interferons due to virus-mediated inhibition of type 1 interferon production. Thus, DAMPs predominate in initiating innate immune activation and systemic inflammation (arrow 3), represented with variable *I*, that will self-upregulate (arrow 4). The effects of inflammation can create collateral host tissue damage (arrow 2), providing positive feedback into *D*. The priming of an adaptive response, *A*, is initiated and upregulated by *I* (arrow 5), and *A* in turn inhibits the upregulation of *I* (arrow 9). If the *I* response becomes strong enough, *I* switches to inhibiting the upregulation of A (arrow 8). The adaptive response, *A*, will also be stimulated in response to PAMP signaling from virus-infected cells, albeit with somewhat delayed kinetics because of viral proteins that attenuate such signaling mechanisms (arrow 6). When encountering virus-infected cells, *A* can kill these cells (*C_V_*) by the action of virus-specific cytotoxic T cells and/or neutralize the virus with antibody secreting virus-specific B cells, thereby inhibiting the expansion of the virus-infected cell population (arrow 10).

To build our mathematical model, we posited that the virus-infected epithelial cells in the respiratory tract signal in a bifurcated manner either *via* PAMPs or through cellular damage and/or dysfunction *via* DAMPs, inducing activation of virus-specific adaptive immune responses *via* innate cell activation (**Figure 1**). Within the network structure, the model considers the interactions of four abstract variables that can be thought of as cellular compartments and their associated molecular processes: 1) Virus-infected epithelial cells, *C_V_*; 2) Damaged/dysfunctional cells/tissue that promote inflammation *via* the release of DAMPs, *D*; 3) Innate Immune/systemic inflammatory components, *I;* and 4) Adaptive Immune components, *A*, which incorporate antigen presenting cells that are part of the innate system (i.e. dendritic cells) and bridge the feed-forward communication between *I* and *A*. The compartmental mechanistic model is a parsimonious representation of the myriad immune and inflammatory events occurring during infection. The details of the model are given in the *Methods* and **Supplementary Methods**.

**Figure 2:**
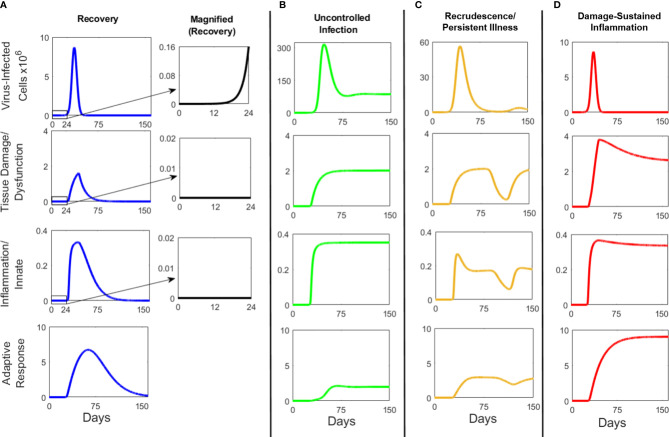
Illustrations of theoretical response dynamics of the SARS-CoV-2 model. Panels **(A–D)** display uncalibrated simulations of model variables *C_V_*, *D*, *I*, and *A* where each column illustrates a response profile of these variables for the indicated archetype. Magnified outsets to the immediate right of Panel A illustrate that a window of time exists during which the virus-infected cell population, *C_V_*, is increasing but the host damage/tissue dysfunction (*D*; Panel **(A)** 2^nd^ row) and innate/inflammatory immune response (*I*; Panel A 3^rd^ row) is negligible. Each simulation set (column) is initiated with the equivalent of one virus-infected cell at time zero (*C_V_* =1; Panels **(A–D)** top row) into a naïve simulated individual, meaning the specific immunity (A; Panels **(A–D)** (Panel **B**), bottom row) to *C_V_* is absent at time zero. Together, each panel (column) of time courses of the model variables represent a potential COVID19 archetype. Initial exposure amounts along with specific, early dynamics related to viral infectivity of cells are largely intractable aspects of disease processes outside of a controlled experiment. Thus, we assume that exposures leading to a productive viral infection will have established, at some time, at least one infected cell, from which other cells will become infected. Assuming larger initial levels of the virus-infected cell population at time zero essentially shift the curves to the left, allowing for a more rapid expansion of the virus-infected cells as well as the ensuing response.

A baseline set of parameter choices (**Table S1**) were motivated by previous work modeling viral infection (38–41) as well as immune inflammatory dynamics, the latter in the context of both sepsis (34) and sterile inflammation (35). Exploration of the parameter space around the baseline set generated simulations that predicted distinctive viral load and immune/inflammatory response trajectories that we term COVID-19 “archetypes”. These distinctive infectious disease dynamics are shown in **Figure 2** and with their diverse presentations in **Figure S1**. Notably, the differences among archetypes were due to changes in only four key parameters (*k_di_*; *x_di_*; *x_iainv_*; and *k_ai_*), while the others remained fixed. Multiple parameter sets led to similar manifestations of a response that fell under one of the four major archetypes. As an example, we chose seven parameter sets to provide a representation of the possibilities, all of these arose from changes made to one parameter set such that the overall differences between the number of parameter values differing from one scenario to the next was at most three. For instance, the pathways in the model that lead from the Mild to the Moderate to the Severe scenarios of the *Recovery* archetype all result from a change in two parameters (namely the parameters responsible for inducing *D via I*: *k_di_* and *x_di_*). This is not the only pathway to achieve this progression (see **Supplementary Materials**).

**Figure 3:**
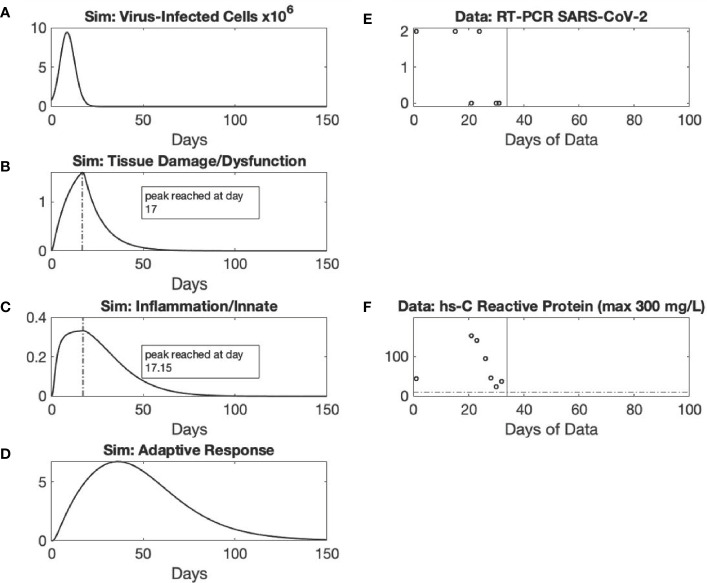
*Recovery* archetype, Mild response scenario: simulation *vs*. patient data. Using the baseline set of model parameter values, simulated dynamics are shown of variables *C_V_* (Panel **A**) (Initialized with a C_V_ =8x10^5^ virus-infected cells), *D* (Panel **B**), *I* (Panel **C**), and *A* (Panel **D**). The following six parameters with their baseline values given are those used in the simulation of the Mild scenario and are the subset of parameters whose values may differ between the Mild scenario and other scenario simulations as specified within **Table S1**: k_di_=0.3; x_di_=1.0, x_iainv_=0.1; k_ai_=3.0; k_ii_=0.1; k_id_=0.35. Panels **(E, F)** show data from a patient in their 60s with hypertension, diabetes, and chronic kidney disease who tested positive for SARS-CoV-2 while asymptomatic, as a result of screening for a planned procedure. The patient had the opportunity to be tested several more times over the ensuing weeks while hospitalized for non-related medical problems. For the entirety of their hospitalization (black vertical lines denote discharge), the patient was not overtly symptomatic for COVID-19.

**Figure 4:**
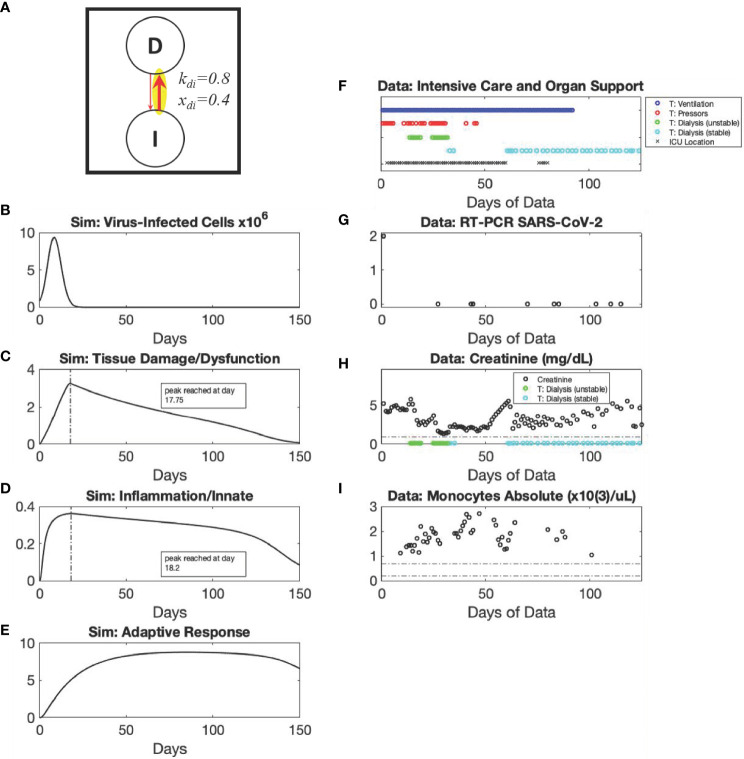
*Recovery* archetype, Severe scenario: simulation *vs.* patient data. Using modified values of parameters k_di_ and x_di_ compared to Mild scenario as shown in the diagram (Panel **A**), simulated dynamics are shown of *C_V_* (Panel **B**) (Initialized with a *C_V_* =8x10^5^ virus-infected cells), *D* (Panel **C**), *I* (Panel **D**), and *A* (Panel **E**). Panels **(F–I)** show data from a patient in their 50s with diabetes who presented with COVID-19 acute respiratory distress syndrome. The patient was most critically ill from hospital day 14 to 32, requiring intermittent vasopressors and being unable to tolerate intermittent hemodialysis (instead requiring continuous venovenous dialysis). Despite being ventilator dependent until Hospital Day 93, the patient was able to leave the intensive care unit by Hospital Day 60. They were discharged to a skilled nursing facility on the 125^th^ day, tolerating a speaking valve (tracheostomy) with a strong voice and walking with assistance but still requiring dialysis.

**Figure 5:**
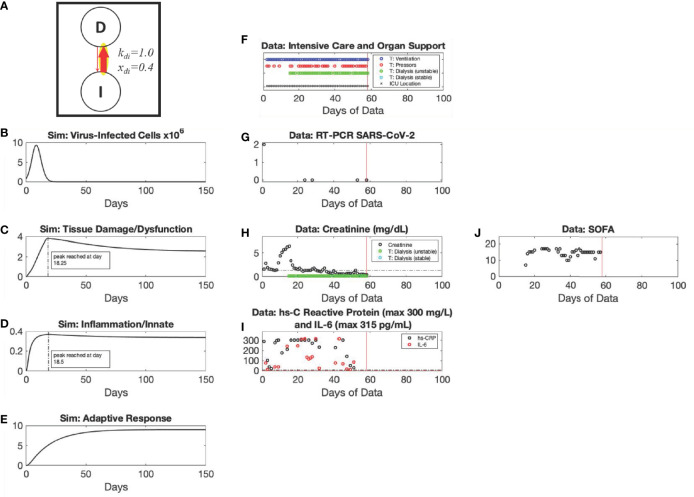
*Damage-sustained Inflammation* archetype: simulation *vs*. patient data. Using modified values of parameters k_di_ and x_di_ compared to the *Recovery* archetype, Mild scenario as shown in the diagram (Panel **A**), simulated dynamics are shown of *C_V_* (Panel **B**) (Initialized with a *C_V_* =8x10^5^ virus-infected cells), *D* (Panel **C**), *I* (Panel **D**), and *A* (Panel **E**). Panels **(F–J)** show data from a patient in their 50s with diabetes who presented with hypoxemia and increased work of breathing after 6 days of shortness of breath and chest pain at home. The patient was found to be in diabetic ketoacidosis and was intubated the day after admission. The patient developed COVID-19 Acute Respiratory Distress Syndrome and their complex course included deep venous thromboses, limb ischemia, vasodilatory shock, pulmonary embolus and cardiogenic shock, and renal failure from COVID-19 requiring dialysis. The patient was given a course of methylprednisolone starting on hospital day 2, and anticoagulated starting on hospital day 7. The patient was given stress steroids from hospital days 29-41 and 44-53. The patient developed cardiac arrest (pulseless electrical activity) on hospital day 34, and on hospital day 44, their hypoxemia and shock worsened. On hospital day 58, the patient went into cardiac arrest and ROSC was achieved after 20 minutes, at which time the patient was noted to have bilateral dilated unreactive pupils. The patient developed pulseless electrical activity and died (red vertical lines).

**Figure 6:**
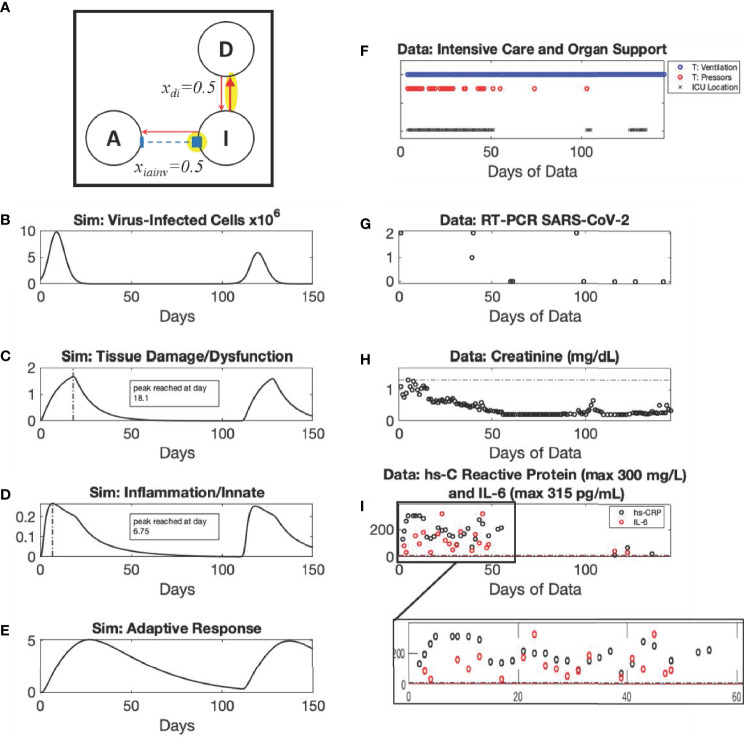
*Recrudescence* archetype: simulation *vs*. patient data. Using modified values of parameters x_iainv_ and x_di_ compared to the *Recovery* archetype, Mild scenario as shown in the diagram (Panel **A**), simulated dynamics are shown of *C_V_* (Panel **B**), (Initialized with a *C_V_* =8x10^5^ virus-infected cells), *D* (Panel **C**), *I* (Panel **D**), and *A* (Panel **E**). Panels **(F–I)** show data from a patient in their 60s with hypertension and no known sick contacts who presented after 4 days of fever, chills, *malaise*, and headache with mild hypoxemia (oxygen saturation 95%) and mild opacities on chest radiograph, found to be SARS-CoV-2 PCR positive. The patient’s oxygen requirement steadily increased over four days and they required mechanical ventilation for acute respiratory distress syndrome. While the patient was still ventilator-dependent, they were able to be de-escalated from the intensive care unit at Hospital Day 50 and required a brief return to intensive care with vasopressor support at day 103. The patient was still ventilator-dependent by the time of their discharge to a long-term acute care (ventilator-capable) facility on hospital day 146.

**Figure 7:**
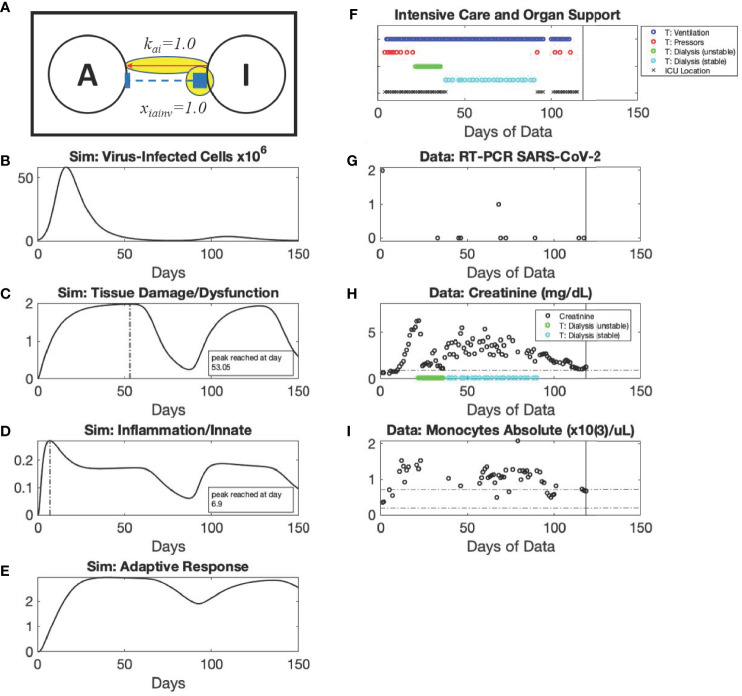
*Recrudescence* archetype, Long-lasting Disease scenario: simulation *vs*. patient data. Using modified values of parameters k_ai_ and x_iainv_ compared to the *Recovery* archetype, Mild scenario as shown in the diagram (Panel **A**), simulated dynamics are shown of *C_V_* (Panel **B**) (Initialized with a *C_V_* =8x10^5^ virus-infected cells), *D* (Panel **C**), *I* (Panel **D**), and *A* (Panel **E**). Panels **(F–I)** show data from a patient in their 60s with diabetes and hypertension who presented with pneumonia and SARS-CoV-2 positivity. The patient’s course had two distinct periods of critical illness. They required intubation by Hospital Day 5, and their course was complicated by vasopressor-dependent hypotension and renal failure requiring continuous venovenous hemodialysis from Hospital Days 23-38. The patient was able to leave the intensive care unit on Hospital Day 39 but returned to intensive care on Hospital Day 92 with new hypotension requiring vasopressors, status epilepticus, and ischemic stroke. The patient was ventilator-independent by Hospital Day 95, recovered renal function, and was able to be discharged to a skilled nursing facility on Hospital Day 118 (black vertical lines).

**Figure 8:**
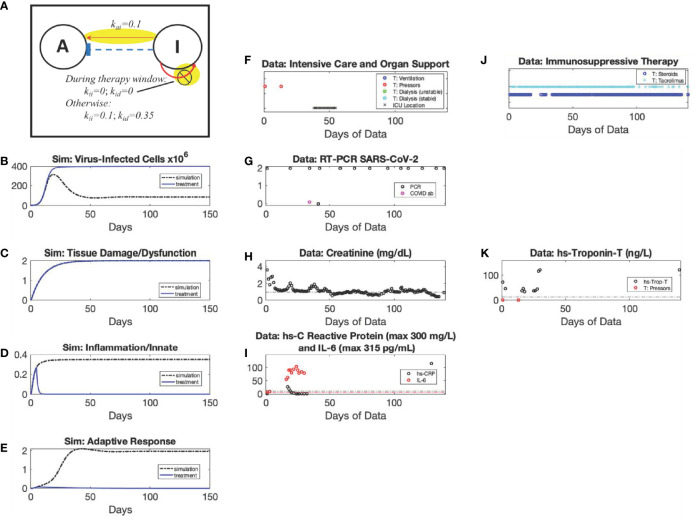
*Uncontrolled Infection* archetype and Long-duration Anti-inflammatory Treatment: simulation *vs*. patient data. The *Uncontrolled Infection* archetype emerges due to modifying the value of parameter k_ai_ compared to the *Recovery* archetype, Mild scenario; and, when implementing the window of anti-inflammatory therapy, k_ii_ and k_id_ are set to zero as shown in the diagram (Panel **A**). Simulated dynamics are shown of *C_V_* (Panel **B**) (Initialized with a *C_V_* =8x10^5^ virus-infected cells), *D* (Panel **C**), *I* (Panel **D**), and *A* (Panel **E**) for the Uncontrolled Infection archetype (black, dot-dashed curves, Panels **B–E**). An anti-inflammatory treatment simulation of this archetype is overlayed (blue, solid curves, Panels **B–E**). Panels **(F–K)** show data from a patient in their 60s who was chronically immunosuppressed after a solid organ transplant who presented after weeks of diarrhea and failure to thrive. The patient was found to be in renal failure and SARS-CoV-2 PCR positive. The patient continued on prednisone for most of their hospitalization course. On hospital day 15, the patient developed their first oxygen requirement and was found to have ground glass opacities on chest computed tomography. The patient was given tocilizumab on hospital day 17 and remdesivir on hospital day 24. They were then transferred to an intensive care unit on hospital day 38 for closer monitoring and more oxygen supplementation (non-rebreather mask), but never required invasive or positive pressure ventilation. The patient was discharged home with health services on hospital day 135.

**Figure 9:**
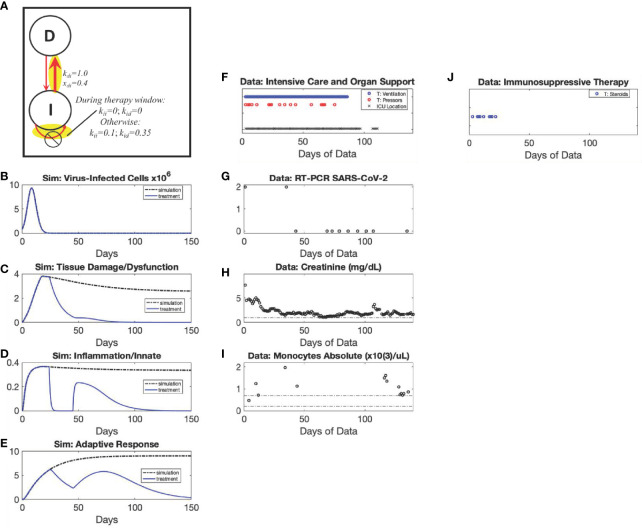
Short-duration Anti-inflammatory Treatment: *Damage-sustained Inflammation* archetype simulation with and without treatment *vs*. patient data. The *Damage-sustained Inflammation* archetype emerges due to modifying the values of parameters *k_di_* and *x_di_* compared to the *Recovery* archetype, Mild scenario; and, when implementing the window of anti-inflammatory therapy, *k_ii_* and *k_id_* are set to zero as shown in the diagram (Panel **A**). Simulated dynamics are shown of *C_V_* (Panel **B**) (Initialized with a *C_V_* =8x10^5^ virus-infected cells), *D* (Panel **C**), *I* (Panel **D**), and *A* (Panel **E**) for the Damage-sustained Inflammation archetype [black, dot-dashed curves, Panels **(B–E)**]. An anti-inflammatory treatment simulation of this archetype is overlayed [blue, solid curves, Panels **(B–E)**]. Panels **(F–J)** show data from a patient in their 50s with hypertension, chronic kidney disease, and obesity who, after two weeks of symptoms at home, presented hypotensive and in hypoxemic respiratory failure. The patient was intubated on hospital day 3 and given a methylprednisolone course on hospital days 7-11 as well as hydrocortisone stress steroids on hospital days 17-19. The patient was liberated from the ventilator by hospital day 86 and discharged to a skilled nursing facility by hospital day 140. Simulated Anti-inflammatory intervention was carried out in the Damage-sustained Inflammation archetype of Figure 5 Panels B-E. By temporarily adjusting some of the model parameter values governing the generation of inflammation, I, the model simulation emulates the results of applying a broad-spectrum anti-inflammatory intervention resulting in massive suppression of inflammation. Treatment in the simulation started on day 24 (when virus-infected cells were still elevated, implying the possibility of a positive PCR result, *C_V_* =1.7x10^4^ cells), where we estimated this by adjusting for the 14 days of symptoms prior to admission plus steroids started at day 7. The patient only received 13 days of treatment; however, our estimated window of 21 days used in the simulation resulted in a favorable outcome as opposed to shorter times investigated (**Figure S4**). The simulated therapy shows that while inflammation is drastically reduced during the treatment window, there is a resurgence after the treatment is stopped (See Panel D, blue curve, and Panel I, Data: Monocytes Absolute) due to the continued presence of inflammation-inducing Damage/Dysfunction (See Panel C, blue curve, and Panel F, Data: Intensive Care and Organ Support). However, the treatment is enough to reset the response dynamics in the simulation so that resolution is achieved by 150 days, having significantly inhibited the subsequent creation of D and stopping the positive *I* to *D* feedback loop.

**Figure 10:**
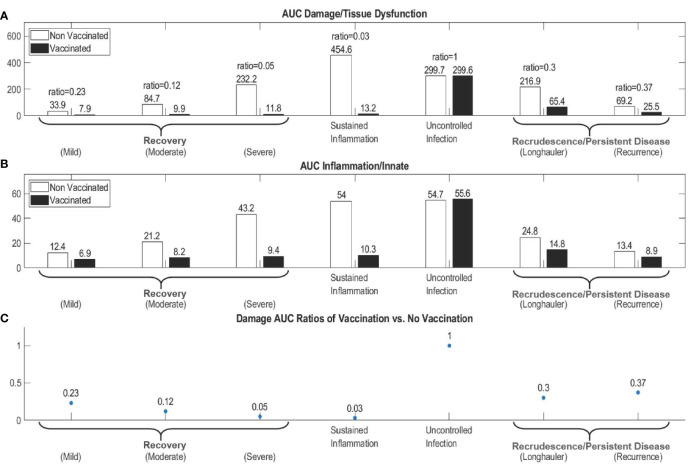
Area under the curve (AUC) calculations for Damage/Tissue Dysfunction and Inflammation/Innate Immunity states in order to compare results of the non-vaccination to the vaccination scenarios. AUC for the Damage/Tissue Dysfunction state, *D*, (Panel **A**) and AUC for Inflammation/Innate Immunity state, *I*, (Panel **B**) in non-vaccination simulations and vaccination simulations are compared for seven scenarios spanning the four archetypes. In (Panel **A**), the ratios of AUC_D_-Vac to AUC_D_-NoVac for each scenario in (Panel **A**) are displayed above the respective pair of bar graphs and quantify the relative effectiveness over all the scenarios of the vaccination in reducing overall damage. These ratios are also graphed in (Panel **C**) Ratios < 1 show effectiveness of vaccination for reducing overall AUC in a scenario, whereas Ratios ≥ 1 indicate that vaccination was not helpful and potentially harmful. For the non-vaccination simulations, AUC is calculated over 160 days post infection, whereas the vaccination simulation AUCs are calculated from the time of the first vaccination dosage plus 160 days post infection, 160 + 42 days, to account for damage created from the adjuvant-inducing *I* response during the vaccination period.

These initial simulations were initiated with a single virus-infected cell, and thus the time to peak infection appeared long. Later, in **Figures 3**–**10** and **S2–S6**, the simulations were initiated with a more realistic viral load corresponding to measurements. Higher viral load shifted all curves to the left, lessening the elapsed time to reach peaks of infection. Importantly the time courses of viral infection and resolution were in reasonable accord with clinical observations (see below), thus providing independent support for the particular set of baseline parameters used for the simulations.

As noted above, differences in the possible outcomes arise from different biologically plausible parameters (**Table S1**). These could represent individual differences in genetic predisposition to infection, propensity for tissue damage/dysfunction, innate immune activation, adaptive immune activation, clinical co-morbidity, infection history, and/or chronic treatments with immunosuppressive agents. We classify the core outcomes into four distinct clinical archetypes: *Recovery*, *Uncontrolled Infection*, *Recrudescence/Persistent Illness*, and *Damage-sustained Inflammation*. Varying manifestations are possible within each archetype. In **Figure 2** the magnified graphs to the right of **Panel A** illustrate that a window of time exists during which the virus-infected cell population is increasing but the host damage/tissue dysfunction and innate/inflammatory immune responses are negligible. We equate this state with asymptomatic COVID-19, in agreement with the finding of radiographic evidence of infection in otherwise asymptomatic COVID-19 patients that do not exhibit signs of systemic inflammation (42). In all archetypes, compartment *C_V_* rises first followed by *D*, *I* and *A*. The difference is in how these variables resolve. In the *Recovery* archetype, the four compartments rise to a peak and then return to low levels. In the *Uncontrolled Infection* archetype, the four compartments rise and remain elevated. In the *Recrudescence* archetype, *C_V_* rises then falls and rises again (possibly multiple times), which is followed by the rise and fall of the three other compartments. Finally, in the *Damage-sustained Inflammation* archetype, the four compartments rise but the *I* and *A* remain elevated even after virus is cleared and *C_V_* has returned to baseline.

The four archetypes of COVID-19 correspond to dynamical attractors (43) of the mathematical model of SARS-CoV-2 infection. All time courses will flow towards one of a finite number of possible attractors that in principle may include stationary equilibria, oscillating cycles, and possibly chaotic dynamics (43). The attractors are fully specified by the network architecture of the immune/inflammatory system but the initial conditions [e.g. viral load (44), background inflammatory status (10, 11, 13, 14, 32)], and parameter settings [e.g. abundance and distribution of ACE2 receptors (45) and possibly also neuropilin-1 (46), as well as immune memory (19)], determine which attractor is selected. Small changes in the initial conditions and parameters can lead to different attractors and thus very different outcomes.

### Model Calibration and Testing: Predicting Realistic Temporal Dynamics of Viral Infection

As an initial calibration and a test of the theoretical model, we sought to accurately recover the temporal dynamics of SARS-CoV-2 infection based on a realistic initial viral inoculum. In a study of SARS-CoV-2 infection in rhesus macaque monkeys (37), the viral peak occurred at approximately day 5 after an initial intratracheal challenge with SARS-CoV-2 at 1x10^6^ 50% tissue-culture infectious doses (TCID_50_). At a starting *C_V_* value of 1x10^6^, our model predicted a peak time for the viral infection at 8 days. An exploration of the impact of increasing initial viral load on the time to peak infection for each COVID-19 archetype is detailed in **Table S2**.

Based on this initial calibration analysis, we next sought to calculate the typical initial *C_V_* value for human COVID-19 patients and, in the context of model verification, compare model variable trajectories to clinical data. This is quite difficult to do based on previously published data [e.g (17)], since those studies are based on patients that were already infected at the time of hospitalization and since data were obtained at only a few time points. We reasoned that granular data on patients hospitalized for reasons other than COVID-19, either with recent negative or initially negative screening tests, that became SARS-CoV-2-positive in the hospital setting (termed “incidental COVID-19”) would address these shortcomings. Accordingly, time of symptoms or PCR positivity data were obtained from “incidental COVID-19” patients hospitalized at New York Presbyterian Hospital (Columbia) between March 13 - August 5, 2020 (**Table 1**).

SARS-CoV-2 infection was ascertained by RT-PCR tests in nasal swabs either as a screening test for procedures or triggered by symptoms including cough or fever. In this cohort, the time to virus positivity was estimated to be 11.3 ± 5.3 (n=8 incidental COVID-19 patients; range 4-22) days. From these data, we calibrated to a *C_V_* peak time of 11.1 ± 4.5 days (n=7 archetypal scenarios; range 8.5-19.1 days) in subsequent simulations based on an initial *C_V_* dose equivalent to 8 x 10^5^ virus-infected cells (**Table S2**), in general agreement with the aforementioned results in non-human primates.

### Model Validation: Temporally Calibrated Theoretical Archetypes Match Individual COVID-19 Cases Qualitatively

We next sought to gain insights into drivers of different COVID-19 responses and validate key predictions of our theoretical model by matching archetypal responses (using simulations of the temporally calibrated model based on a simulated inoculum (*C_V_*) of 8 x 10^5^ virus-infected cells) to clinical phenotypes in our patient cohort. These simulations were based solely on altering model parameters (See **Table S1**) associated with the actions of *D*, *I*, and *A* but without invoking differences in viral dynamics and without fitting directly to the clinical data. We compared the model variables to clinical data as follows: *C_V_* was compared to the results of serial RT-PCR tests. We note categorical RT-PCR data were used; for all patients, categorical determinations of absence of virus (0), a possible positive result (1), or a certain positive result (2) were available. *D* was compared with creatinine (biomarker of kidney function) and to high-sensitivity troponin T (biomarker of cardiac injury). Additionally, Sequential Organ Failure Assessment [SOFA] score (which encompasses organ function across 6 organ systems; this was available only for some intensive care unit (ICU) patients), and ventilatory dependence (surrogate of respiratory distress), were used when available for patients; and *I* was compared to serial measurements of high-sensitivity C-reactive protein [a global but fairly nonspecific biomarker of inflammation (47) that is also a strong predictor of COVID-19 (48, 49)], the cytokine interleukin (IL)-6 [also shown to be elevated in COVID-19 patients (17, 18, 50)], and absolute monocytes (automated)[another hallmark of COVID-19 (51)]. In the *D* and *I* comparisons, we focus on the initial rise of the respective variables. This is because *D* and *I* represent the net action of multiple interacting mediators and thus a biological marker associated with the initial response may not be the one associated with the later stages of the response. The essential elements of the *D* and *I* dynamics with respect to novel viral infection is that *D* activates *I* followed by positive feedback within and between the two compartments. Because *A* is a composite variable that encompasses antigen-presenting cells and the priming of helper CD4 and cytotoxic CD8 T cell responses as well as antibody responses of B cells, and because it is still unclear which of these variables is the one that is involved in reducing viral infection (and when), we did not attempt to directly compare this variable to a marker. We note, however, that key aspects of *A*, such as antibodies to SARS-CoV-2, are very highly correlated with viral infection (52). **Table S3** gives the descriptions of the various scenario labels under the four archetypes used in this manuscript.

The *Recovery* archetype encompasses various manifestations ranging from mild disease course that does not require ICU stay (**Figure 3**) to severe disease course with prolonged ICU stay (**Figure 4**) (an intermediate, moderate scenario is shown in **Figure S2**). In the Mild scenario, viral infection (**Figure 3A**) leads to tissue damage/dysfunction (**Figure 3B**), which only then leads to systemic inflammation (**Figure 3C**), a predicted rise in adaptive immunity (**Figure 3D**), and ultimately to recovery. In support of model simulations, PCR cycle time is seen on the decline in the exemplar patient [**Figure 3E**] with asymptomatic COVID-19, and the return of C-reactive protein to baseline [**Figure 3F**]). This is also seen in both the Moderate (**Figure S2**) and Severe (**Figure 4**) scenarios, which are modeled by altering parameters k_di_ and x_di_ compared to the Mild scenario (**Figure 4A**). The exemplar patient for the Severe scenario became severely critically ill after the peak viral load (**Figure 4G**), requiring ventilatory and circulatory support as well as renal replacement therapy (but was unstable and required continuous venovenous dialysis) (**Figure 4F**). The clinical course improved over the very lengthy hospitalization, with the patient being able to transition out of the intensive care unit, off of vasopressors and tolerating intermittent hemodialysis; eventually, this patient was liberated from the ventilator (**Figure 4F**). The markers of inflammation were high and elevated for nearly the entire length of hospital stay, with some decline towards the end (**Figures 4H, I**).

In these scenarios of the *Recovery* archetype, viral infection is cleared; recovery ultimately occurs if the innate-to-adaptive immune response is sufficiently effective, and the resulting damage is not too severe. The duration and severity of the episode can vary greatly depending on how strongly *C_V_*-induced damage incites an inflammatory response and, in turn, how strongly that response upregulates more inflammation, providing subsequent *I*-induced damage in the positive feedback cycle. Despite the explicit modeling of *D* before *I*, our theoretical model predicts, and clinical data support, the notion that the peak of *D* will be fairly close to the peak of *I* (and possibly indistinguishable from the possibility that *C_V_* leads initially to *I* rather than to *D*) as the response to viral infection becomes more pronounced and leads to increased disease severity requiring ICU stay (**Figures 4** and **S2**). Specifically, despite an initially earlier and much faster rise of *D* relative to *I* (**Figure S3**), this distinction is essentially undetectable by Day 1.

If the innate immune response and attendant systemic inflammation causes too much secondary damage/dysfunction (simulated by modifying values of parameters *k_di_* and *x_di_* compared to Mild scenario [**Figure 5A**]), then the positive feedback loop between *D* (e.g. by the release of DAMPs) and *I* can lead to a condition of *Damage-sustained Inflammation* as shown in **Figure 5**. The exemplar patient for this archetype was critically ill, requiring ventilatory and circulatory support for the entirety of their course, ending in death (**Figure 5F**). The creatinine course (renal disease/damage biomarker) appears normalized (**Figure 5H**) as a consequence of dependence on renal replacement therapy; however, this patient was too hemodynamically unstable even for intermittent hemodialysis and their SOFA score remained high (**Figure 5J**). Inflammation biomarkers (CRP and IL-6) were high and elevated for the entirety of their course (**Figure 5I**). Here, our modeling suggests (**Figure 5B**) and the RT-PCR data support (**Figure 5G**) the notion that the adaptive immune system clears the virus successfully, but inflammation persists until the patient finally succumbs or is controlled by medical intervention. Additionally, since the adaptive immune response as modeled incorporates the role of dendritic cells (and potentially other antigen-presenting cells) which represent the mechanism by which *I* primes *A*, the adaptive immune response can appear to remain elevated (**Figure 5E**) even when virus has been cleared (below 1 virus-infected cell at approximately day 35 of the simulation) (**Figure 5B**) and is no longer providing stimulus feedback to *A* for expansion. This is in line with recent studies (53). Our simulations suggest that this is due predominantly to the persistent presence of activated antigen-presenting cells rather than activated virus-specific B and T cells; recent findings (54) support these simulation results. We note that the antigen-presenting cells were included in the *A* rather than the *I* variable though these cells could arguably be included in either variable (depicted for all archetypes/scenarios **Figure S4**, insets).

The sustained inflammation state has often been compared to a “cytokine storm” in sepsis (32, 55), but we note that the actual levels of circulating inflammatory mediators need not be elevated excessively but instead are simply sustained over a prolonged period, as has been reported by others (56) (compare peak levels of *I* and *D* in **Figures 3** or **4** [*Recovery* archetype] *vs*. **Figure 5** [*Damage-sustained Inflammation* archetype], as an example). In the *Damage-sustained Inflammation* archetype, antiviral therapies will be ineffective in mitigating sustained inflammation, but anti-inflammatory therapies such as corticosteroids may help [although timing and duration is important (see below)]. Therapies that mitigate damage/dysfunction, most likely organ-supportive care in the ICU, as well as DAMP-targeted therapies (28, 29) such as those described in the setting of bacterial sepsis (57), might also be effective for the *Damage-sustained Inflammation* archetype of COVID-19.

The *Recrudescence* archetype (**Figures 6**, **7**) can arise if the adaptive immune response diminishes before the virus is eliminated completely. This state can appear under widely divergent dynamical scenarios [simulated by modifying values of parameters *x_iainv_* and *x_di_* compared to the Mild scenario (**Figure 6A**) or parameters *k_ai_* and *x_iainv_* compared to the Mild scenario (**Figure 7A**)]. This archetype can arise simply because the adaptive response abates too quickly, or because the feedback mechanisms among the compartments give rise to oscillations that can either dampen or grow and thus lead to a healthy resolution or eventual death. Dynamically, there is little difference between recrudescence and reinfection. In both cases, the virus has a chance to regrow either by returning from low levels from a hidden reservoir in the body or by reintroduction through an exogenous source. The exemplar patient in **Figure 6** has two periods of critical illness (the first more severe than the second, **Figure 6F**) that co-occur with recurrent viral detection (**Figure 6G**).

The *Recrudescence* archetype in **Figure 7** displays a disease course that is reminiscent of individuals with long-lasting disease [a condition colloquially referred to as “long COVID” (7–9, 58)]. In this representative scenario, it is possible that the predicted second peak of viral infection (**Figure 7B**) occurs at levels that are just at the threshold of detection of current nasal swab RT-PCR tests (**Figure 7G**). The exemplar patient was critically ill, requiring circulatory and respiratory support followed by renal replacement therapy (**Figure 7F**). They were able to be weaned off pressors and tolerate intermittent hemodialysis and eventual renal recovery, leaving the ICU for a long period of time (**Figure 7F**). However, they experienced a second rise in inflammation (monocyte counts shown in **Figure 7I**) as the second (low) viral load is detected (**Figure 7G**), followed closely by a second period of critical illness requiring a shorter period of circulatory support in the context of seizures and a stroke (**Figure 7F**).

If adaptive immunity is not activated sufficiently or is not effective enough in clearing virus-infected cells, then the *Uncontrolled Infection* archetype can result, as seen in **Figure 8** (where this was simulated by modifying values of parameter k_ai_ compared to the Mild scenario; **Figure 8A**). Such an archetype might occur naturally [e.g. in obese individuals (59)]. An alternative scenario could be that of an immunocompromised individual or a transplant patient who is receiving chronic immunosuppressive therapy. This latter scenario was the case in the exemplar patient who was SARS-CoV-2 positive on admission but developed model-predicted (**Figure 7C**) mild renal failure (which resolved; **Figure 8H**) and an oxygen requirement (which also resolved; **Figure 8F**) related to COVID-19 two weeks into admission. Notably, virus was detectable for the entirety of the lengthy hospitalization (**Figure 8G**). In this state, the simulations (**Figures 8B–E**) show that all model variables are elevated, and thus small changes in model parameters due to therapeutic effects could redirect the system from uncontrolled infection into sustained inflammation. Therefore, guiding such a condition back to a healthy state could require a dynamic therapy that constantly titrates between controlling the infection and the inflammatory response (60, 61). For example, the exemplar patient received the anti-viral drug remdesivir (62, 63) and yet remained virus-positive (**Figure 8G**).

### Model Validation: Simulating Anti-Inflammatory Therapy

We validated our model by recapitulating core features of the response to the administration of corticosteroids, a widely used anti-inflammatory therapy for COVID-19 that has to date been the only one considered efficacious based on a meta-analysis of clinical trial results (64). The exemplar patient in **Figure 8** received corticosteroids from admission until day 135 (with a brief interruption from days 18-24, 26, 30-33; and 65). We estimated the therapy window for our simulation to be continuous from day 5 (to allow for the simulated response to evolve from the initial conditions) through day 160. Corticosteroid treatment was simulated as being effective at suppressing inflammation by fully suppressing all inflammatory induction pathways in the model (i.e. parameters k_ii_ and k_id_ in the *I* equation were set to zero during treatment windows). Anti-*I* treatment for this archetypal patient shown in **Figure 8J** suppresses *I* to levels that resemble a mild recovery case; however, in this *Uncontrolled Infection* archetype, this inhibits *A* from curbing the growth of *C_V_*, which reaches high, sustained levels (**Figure 8B**, blue solid curve) above the scenario without treatment (**Figure 8B**, dot-dash curve). Damage, although relatively low, remains sustained as well (**Figures 8C, H, K**). The therapy did not curb virus growth (**Figures 8B, G**), which could lead to complications later. Indeed, the patient was discharged while stable (oxygenating well on room air) but frail with daily nursing care and a palliative care plan.

We next sought to determine if our model could also reproduce the response to a shorter course of anti-inflammatory therapy, in this case a patient in their 50s with hypertension, chronic kidney disease, and obesity who developed severe acute respiratory distress syndrome due to COVID-19 and was administered corticosteroids. Based on the clinical characteristics of the patient, we simulated the administration of corticosteroids to the *Damage-sustained Inflammation* archetype presented in **Figure 5**. Several short-term anti-*I* treatment implementations were explored in which the duration of the treatment lasted for a 14- or 21-day period and a variety of treatment start times were chosen. **Figure 9** presents the data of the patient described above in comparison with simulation results of one of these implementations, in which treatment was initiated *in silico* on Day 24 and lasted 21 days. The treatment was considered successful, namely due to the lower levels of virus-infected cells at the time of treatment compared to peak (*C_V_* =1.7x10^4^ *vs*. *C_V_* =9.3x10^6^ cells at peak). However, a similar implementation in which *in silico* 21-day corticosteroid therapy was delayed until Day 36 displayed a longer recovery and larger area under the curve for *D* (AUC_D_) over the time window compared to results with treatment started on Day 24 (AUC_D_=140 *vs*. ACU_D_=100.3, respectively). **Figure S5** shows this alternative implementation along with all the other theoretical implementations of corticosteroid therapy as an illustration of both successful and unsuccessful interventions, illustrating that the patient represented in **Figure 5** may have benefited from a different administration of corticosteroids.

### Model Prediction: Simulating Vaccination

Multiple COVID-19 vaccines are currently being deployed worldwide (65). As a further test of our underlying hypothesis regarding a role for DAMPs in driving immune responses to SARS-CoV2, and to further validate our model, we next simulated the responses to vaccination of the predicted COVID-19 archetypes. We simulated vaccination in a realistic fashion, by initiating an adjuvant-inducing dose at Day 0 and then again at Day 28. This type of vaccination is assumed to induce *I* to subsequently prime *A* in naïve individuals not previously exposed to SARS-CoV-2 (i.e. absent immune memory). The crucial difference of the vaccine relative to viral infection is that it induces innate inflammatory pathways (*I*) directly without requiring the induction of damage (*D*) to prime a virus-specific adaptive response (*A*). Thus, vaccination in theory provides a safe route for inducing immunity. Although the ensuing immune response may cause symptom-like side-effects, the amount of damage caused by this adjuvant-induced immune response (i.e. *I* ➔*D via k_di_*) are much reduced compared to those caused by the virus.

A concern we addressed initially was that vaccination would occur in an environment in which SARS-CoV-2 infection is likely to occur shortly after vaccination and before any onset of true immune memory. Accordingly, an infection was initiated with the equivalent of 8 x 10^5^ infected cells 14 days following the second vaccination dose. In the simulations, the strength of the adjuvant to induce *I* as well as the effectiveness of the specific antibody response can be adjusted to inhibit the growth of the virus-infected cell population, *C_V_*. The matching control, non-vaccination scenarios presented assume only cell-mediated killing of *C_V_*. Thus, the vaccination protocol enables *A* to both inhibit growth of *C_V_* as well as to kill *C_V_* directly (See **Supplementary Methods** for further details). The development of long-term memory is not considered in the vaccination simulations as it is still unclear how long, and by what mechanisms, this takes to develop in individuals who have been exposed to SARS-CoV-2.

**Figures S6A–G** depict the time courses in each of the core COVID-19 archetypes for both non-vaccination *versus* vaccination simulations. Qualitatively, most of the overall outcomes are unchanged with vaccination except for the *Damage-sustained Inflammation* archetype and the Recurrence scenario of the *Recrudescence Archetype*, which are both converted to a *Recovery* archetype. To further compare between non-vaccination and the vaccination simulations, we calculated the area under the curve of *D* (AUC_D_-Vac, AUC_D_-NoVac) and *I* (AUC_I_-Vac, AUC_I_-NoVac) as well as the ratios of the AUC_D_-Vac to AUC_D_-NoVac for each of the core archetypes (**Figure 10**). Scenarios with an AUC_D_ ratio < 1 demonstrate that the vaccination reduced the amount of overall damage during the simulation time period; the smaller the ratio, the more effective the vaccination was in reducing overall damage/dysfunction. Based on this analysis, we suggest that scenarios related to the *Damage-sustained Inflammation* archetype would benefit most from vaccination, followed by those of the *Recovery* archetype (in reverse order of scenario severity) and then those of the *Recrudescence/Persistent Disease* archetype, especially the recurrence scenario which was converted to recovery under vaccination simulation. For the ratios ≥ 1, this implies that the vaccination was not effective at reducing overall damage, such as in the *Uncontrolled Infection* archetype. Ratios much greater than 1 would imply that vaccination may be harmful, but this was not observed in the simulations.

We next simulated vaccination in the context of fully developed immune memory (implemented as a single parameter rather than adding to the model detailed mechanisms of the formation of immune memory). We experimented with varying levels of an existing level of *A* in the model, implemented as a source constant, A_e_, in the *A* equation (data not shown) and settled upon A_e_=0.05 *A*-units as a reasonable intermediate level of existing immunity based on the dynamics of *C_V_*. The results (**Figures S6H–N**) suggest that fully established immunity is effective at improving the overall outcome of all scenarios compared to the simulations with short-term immune memory (**Figures S6A–G**), as all scenarios/archetypes remained or were converted to a recovery archetype with existing immunity.

## Discussion

The overarching hypothesis of this study was that the SARS-CoV-2-mediated delay in pathogen-associated molecular pattern (PAMP) response that includes diminished type I interferon signaling, shifts the balance of the virus induced immune response to one that is dictated by damage-associated molecular pattern (DAMP) molecules. We reasoned that the current paradigm regarding the sequence of events that ensue in response to viral infection might miss a crucial role for DAMPs released secondary to tissue damage/dysfunction following viral infection, as opposed to the current dogma which focuses on virus-derived PAMPs as the main initiating response. A parsimonious mathematical model, which is based on the overarching hypothesis, revealed that this DAMP-dominated response can manifest different dynamics i.e. result in distinct viral load and disease trajectories that are referred to as archetypal COVID-19 responses or “COVID-19 archetypes”. Importantly, alterations of interactions among virus-infected cells, the magnitude of the innate immune response to DAMPs that are released from damaged or dysfunctional tissue, and the ensuing adaptive immune response triggered by innate immune activation are shown to underlie the distinct disease dynamics or archetypes.

Multiple groups have generated mechanistic (66–68) and data-driven (17, 69–71) computational models of SARS-CoV-2 infection and COVID-19 progression. In our study, we extend this prior work by treating severe COVID-19 disease as a form of critical illness (72). Accordingly, we sought to leverage prior mechanistic mathematical modeling studies in other contexts of critical illness such as sepsis (34, 73–75) and trauma (76–78), all of which incorporate a variable that accounts for tissue damage/dysfunction (*D*) as a proxy for DAMP release. Notably, these earlier models do not include any variables accounting for adaptive immunity, and yet still predicted multiple, archetypal responses to infection or injury. Similarly, our theoretical model predicts distinct COVID-19 archetypes including mild scenarios, and data from incidental COVID-19 patients reflect these predictions.

The hypothesis that *D* is a dominant instigator of *I* in COVID-19 is a novel explanation for why asymptomatic cases are so prevalent in such a deadly disease. Symptoms such as fever and malaise arise after *I* becomes sufficiently high. In the setting of viral infections for which there is immune memory, *I* is activated before or simultaneously with *D* and thus symptoms appear rapidly. With SARS-CoV-2 infection, *I* is induced much later at least in part due to virus-mediated suppression of anti-viral mechanisms (15, 18, 22–26), and thus individuals can have detectable virus in the absence of symptoms. Interestingly, as our simulations progress from mild to more severe COVID-19, the peak of *I* appears much closer to the peak of *D*, and this might explain why the current paradigm would emerge in the absence of extensively granular data on key molecular and cellular players. In these studies, we compared *D* to clinical data elements (e.g. creatinine or SOFA score). However, the molecular identity of *D* in the context of COVID-19 (which, in our model, stimulates *I*), is as yet unknown, though some have speculated that HMGB1 might play this role (28, 29). IL-33 is another key DAMP whose levels are predictive of severe COVID-19 disease and poor outcomes (79).

A property of dynamical systems such as the mathematical model of SARS-CoV-2 infection presented herein is that the time courses flow to attractors, which can include stationary equilibria, oscillating cycles, and even chaotic dynamics (43). The model has an unstable equilibrium at which *A* is at a low positive value while the other three compartments are zero, which is not a dynamical attractor. The introduction of any amount of *C_V_* will take the system out of this unstable equilibrium and into an attractor. The attractors for the *Recovery* and *Recrudescence* archetypes are oscillating cycles. The difference between the two archetypes is the period and dynamic “shape” of the cycle. In the *Recrudescence* scenario, the growth of *C_V_* induces *D*, which in turn induces *I* and *A*, which then suppresses *C_V_*. This causes a reduction in *D*, *I*, and *A*. If these variables decay too quickly, then *C_V_* can increase again and the cycle repeats. There are two ways this cycle can be eliminated and allow for the recovery of the patient. The first is that the period is infinitely long or at least longer than the lifespan of the patient, and this suppresses *C_V_* to zero and keeps it there. This behavior could be due to a long-lasting adaptive immune memory. The second is that *C_V_* is suppressed to zero. While suppression to zero cannot occur in the current differential equation model by design, it could occur if the innate stochastic fluctuations of the discrete molecule interactions were included. When the numerical values of variables are high, these fluctuations are small and do not affect the dynamics; however, when *C_V_* is suppressed sufficiently, these neglected fluctuations could take *C_V_* to zero and eliminate *C_V_* completely. Thus, in some borderline cases, the virus could either be cleared completely or regrow due purely to random chance. In support of this assertion, these possibilities (*Recovery vs*. *Recrudescence*) are observed in controlled studies of SARS-CoV-2 infection in rhesus macaque monkeys (37).

The attractor for the *Damage-sustained Inflammation* archetype is one in which both *D* and *I* flow to an equilibrium at which they both are elevated due to the positive feedback within and between the two compartments. A characteristic of dynamical systems is that widely disparate network architectures can lead to the same attractor. For concreteness, we selected one possible form, but there are multiple ways that the two compartments can be coupled such that the same sustained inflammation attractor will exist. Thus, while our model may not accurately describe the actual mechanisms for specific inflammatory and damage markers *per se*, it captures the emergent attractor dynamics of the system, for which a large family of mechanisms would follow. The attractor for uncontrolled infection is a stable equilibrium at which all the compartments have high values. In this equilibrium, *A* cannot diminish *C_V_* and thus *C_V_* saturates at a high value and keeps the other variables high as well.

Interventions such antiviral or anti-inflammatory drugs can switch the system from one attractor to another or change the shape of an attractor, but do not eliminate or add attractors. Anti-inflammatory therapies may also not be effective because *D* will still be present and sustain the inflammatory state, which will eventually lead to death. To escape from this attractor, a coordinated diminution of both *D* and *I* is necessary. However, if a person is in a state of uncontrolled infection, then antiviral drugs would be effective. One counter-intuitive hypothesis is that pro-inflammatory stimuli might be beneficial; this has been suggested previously in other forms of critical illness, such as sepsis (60, 61) and trauma (80).

This complexity may help explain the conflicting studies about purported antiviral drugs such as remdesivir: a large initial study noted beneficial effects (62), but more recent data suggested no benefit (63). This attractor diagram may also explain the variable effects of broad-spectrum anti-inflammatory drugs such as corticosteroids. In a case where *I* is suppressing *A*, the dose of the anti-inflammatory drug needs to be titrated so that *I* is reduced enough to no longer suppress *A* but not so much that it does not activate *A* at all; this may be one reason why only 1 out of 8 COVID-19 patients responded well to dexamethasone in a recent large trial (81). Our own simulations of corticosteroid administration suggest that even relatively similar timing and duration of therapy applied to the same archetype can result in different overall outcome as well as durations to recovery, in line with clinical experience. Ultimately, we believe that model-based control theory, which exploits the geometric properties of the dynamics are likely to be necessary for addressing the complexity of COVID-19 and other forms of critical illness (82, 83).

Our simulations also offer a nuanced and archetype-specific view into COVID-19 vaccination. Realistic simulations of primary vaccine responses, based on an initial activation of innate immunity due to innate immune sensing of RNA, show that vaccinated individuals will initially undergo an acute inflammatory response to the RNA itself. While our simulations did not point to any setting wherein this inflammation would reach a level similar to that of any of the archetypes associated with severe COVID-19, we cannot rule out individual circumstances wherein this inflammation could trigger inflammatory pathology. Importantly, once adaptive immunity is induced sufficiently, our simulations suggest that overall damage/dysfunction will be reduced in most patients with less severe or recrudescent/persistent disease manifestations, the finding that some archetypes (e.g., *Uncontrolled Infection*) predicted to die in the absence of vaccination will not be rescued by vaccination is sobering. However, this prediction is for individuals that are infected shortly after vaccination, a potentially likely scenario in the context of “pandemic fatigue” and a desire to return to normal activities following vaccination. Our simulations suggest that given sufficient time for an appropriate degree of immune memory to take hold, all COVID-19 archetypes will be protected. These findings would suggest the need to remain vigilant for 1-3 months after vaccination. Notably, our simulations are not directly capable of addressing the issue of further virus transmission by vaccinated individuals.

There are multiple other limitations to this study. Due to its parsimonious nature, the mathematical model described herein does not incorporate details of specific cellular components, their activation states, and discrete molecular pathways, which are aggregated into the broad categories of *I*, *A*, and *D*. This parsimony also means that the model cannot be calibrated directly to data on the dynamics of infection in individual patients. We do not consider this a flaw as much as a necessary tradeoff when the goal is to understand and possibly predict the evolution of infection and immunity in subgroups of patients. As we demonstrate, the model can be calibrated for initial number of virus-infected cells and, in the process, yield quite a good qualitative match to clinical and biomarker trajectories in COVID-19 patients. We note that multiple parameter sets can lead to similar manifestations of a response that fall under one of the four major archetypes presented herein, which can limit the ability to pinpoint the role of specific biological interactions. Another important limitation to the studies presented herein (but not to the model *per se*) is that the model was calibrated to the initially described strain of SARS-CoV2; the multiple variants of this virus that have emerged (84) differ with regard to degree of infectivity and rate of replication, properties that can be modeled readily using our framework. The model also does not account for extra-pulmonary manifestations of COVID-19 and the possibility that the virus infects sites other than the respiratory tracts. We have also not explored every possible therapy, nor have we simulated ICU care *per se*. While the patients exemplified in our study exhibit viral and disease dynamics that support our overall hypothesis, we note that studies using unbiased machine learning approaches with a large cohorts of temporally profiled COVID-19 patients will be needed to fully validate our hypothesis. Notably, our studies also point to the need to assess a role for DAMPs in the diagnostic molecular panels as these could serve as important predictors of disease severity and dynamics.

A key concept in the field of complex systems is that of tradeoffs (85–88). Our studies suggest a novel tradeoff in the context of viral infection and immunity, in which the degree to which type I interferon production is delayed, and thus the extent to which virus-specific host damage/dysfunction is induced, plays a crucial role in linking innate and adaptive immunity and downstream pathophysiology. Ultimately, we suggest that our model is generalizable to other viral infections, with the main tradeoff being the degree to which *I* is initially delayed and the amount of *D* induced by *C_V_*. We posit that seasonal influenza might lead to a smaller degree of *D* as compared to SARS-CoV-2 (with the tradeoff of low individual morbidity but concomitantly low immunogenicity and hence wider spread) as compared to infection with SARS-CoV-2, in line with published findings (89). This may be linked to the consensus that corticosteroids do not improve outcomes in the context of influenza infection (90–92): we suggest that influenza infection generally results in less *D* and hence less *I* as compared to SARS-CoV-2 infection. Inhibiting *I* with corticosteroids in the case of influenza infection may benefit some patients (in cases where the I ➔ D ➔ positive feedback results in elevated inflammation) while harming those patients in which this feedback is not predominant (and thus inhibiting *I* with corticosteroids results in inhibition of *A* and consequent immunosuppression) with a population-level outcome of no net benefit. In the context of SARS-CoV2 infection, we hypothesize that corticosteroids help ~30% of patients (64) at least in part due to direct (*via* inhibition of *D* ➔ I ➔ D) or indirect (by attenuating the negative feedback of *I* on *A*) effects. In contrast, SARS-CoV, MERS, Ebola, or Marburg virus infection would lead to a much larger *D* (with very high individual morbidity and mortality but one that is inherently self-limiting). This feature of self-limiting infection may also be part of the reason that the OC43 coronavirus today causes the common cold despite having been a cause of a pandemic in the 19^th^ century (93). Thus, this feature of SARS-CoV-2-induced *D*, namely that seems to have evolved to be not too big yet not too small, may be the fundamental reason that SARS-CoV-2 has resulted in the COVID-19 pandemic.

## Data Availability Statement

The raw data supporting the conclusions of this article will be made available by the authors, without undue reservation.

## Ethics Statement

The studies involving human participants were reviewed and approved by The institutional review board at Columbia University Irving Medical Center (protocol AAAT0120). Written informed consent for participation was not required for this study in accordance with the national legislation and the institutional requirements.

## Author Contributions

JD conceived the study, performed computational studies, and wrote/edited manuscript. SP conceived the study, obtained and analyzed data, and wrote/edited manuscript. BR obtained and analyzed data, and edited manuscript. HS conceived the study, analyzed data, and wrote/edited manuscript. CC conceived the study, performed computational studies, analyzed data, and wrote/edited manuscript. YV conceived the study, analyzed data, and wrote/edited manuscript. All authors contributed to the article and approved the submitted version.

## Funding

YV was supported by NIH grant U01EB021960. SP was supported by NIH grant R21NS113055. CC was supported by the Intramural Research Program of the NIH, NIDDK.

## Conflict of Interest

YV is a co-founder of, and stakeholder in, Immunetrics, Inc.

The remaining authors declare that the research was conducted in the absence of any commercial or financial relationships that could be construed as a potential conflict of interest.

## Publisher’s Note

All claims expressed in this article are solely those of the authors and do not necessarily represent those of their affiliated organizations, or those of the publisher, the editors and the reviewers. Any product that may be evaluated in this article, or claim that may be made by its manufacturer, is not guaranteed or endorsed by the publisher.

## Abbreviations

hs-CRP: high-sensitivity C-Reactive Protein; Ct: cycle time; CVVDialysis: continuous venovenous dialysis; DAMP: damage-associated molecular pattern; ICU: intensive care unit; iHDialysis: intermittent hemodialysis; IL: interleukin; PCR: polymerase chain reaction; SARS-CoV-2: severe acute respiratory syndrome coronavirus 2; Sim: simulation; SOFA: Sequential Organ Failure Assessment.
