# Supplementary material for: Divergent COVID-19 Disease Trajectories Predicted by a DAMP-Centered Immune Network Model
Source: Front Immunol. 2021 Oct 28;12:754127. doi: 10.3389/fimmu.2021.754127 (PMC8582279; doi:10.3389/fimmu.2021.754127)
Supplement: Supplementary file 1 [file DataSheet_1.pdf]

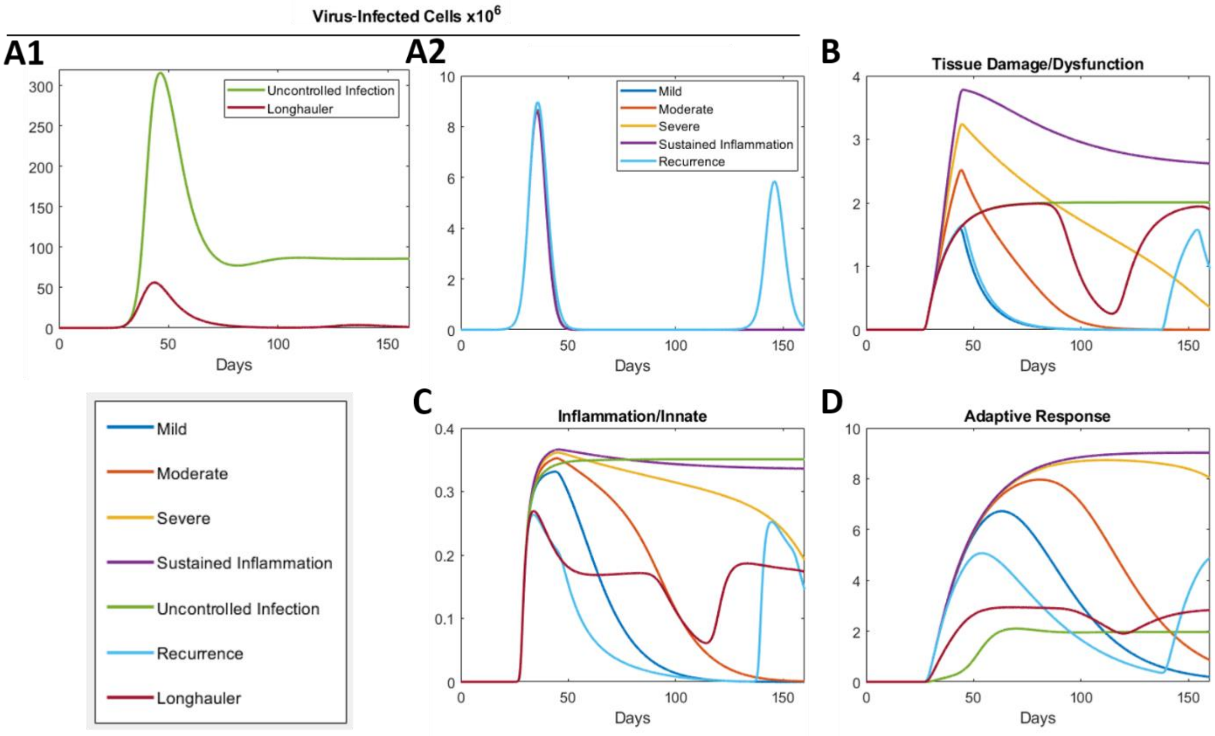

Fig. S1: Composite view of seven scenarios in the four theoretical COVID-19 archetypes. Simulated dynamics of C_V (**Panels A1 and A2**; Initialized with a $C_V = 8x10^5$ virus-infected cells and divided in two panels to assist in viewing), *D* (**Panel B**), *I* (**Panel C**), and *A* (**Panel D**). Simulations are initiated with the equivalent of one virus-infected cell at time zero ($C_V = 10^{-6}$) into a naïve individual, meaning the specific immunity (*A*) to C_V is absent at time zero. A set of the time courses of each variable according to the legend represent a scenario within a potential COVID19 archetype.

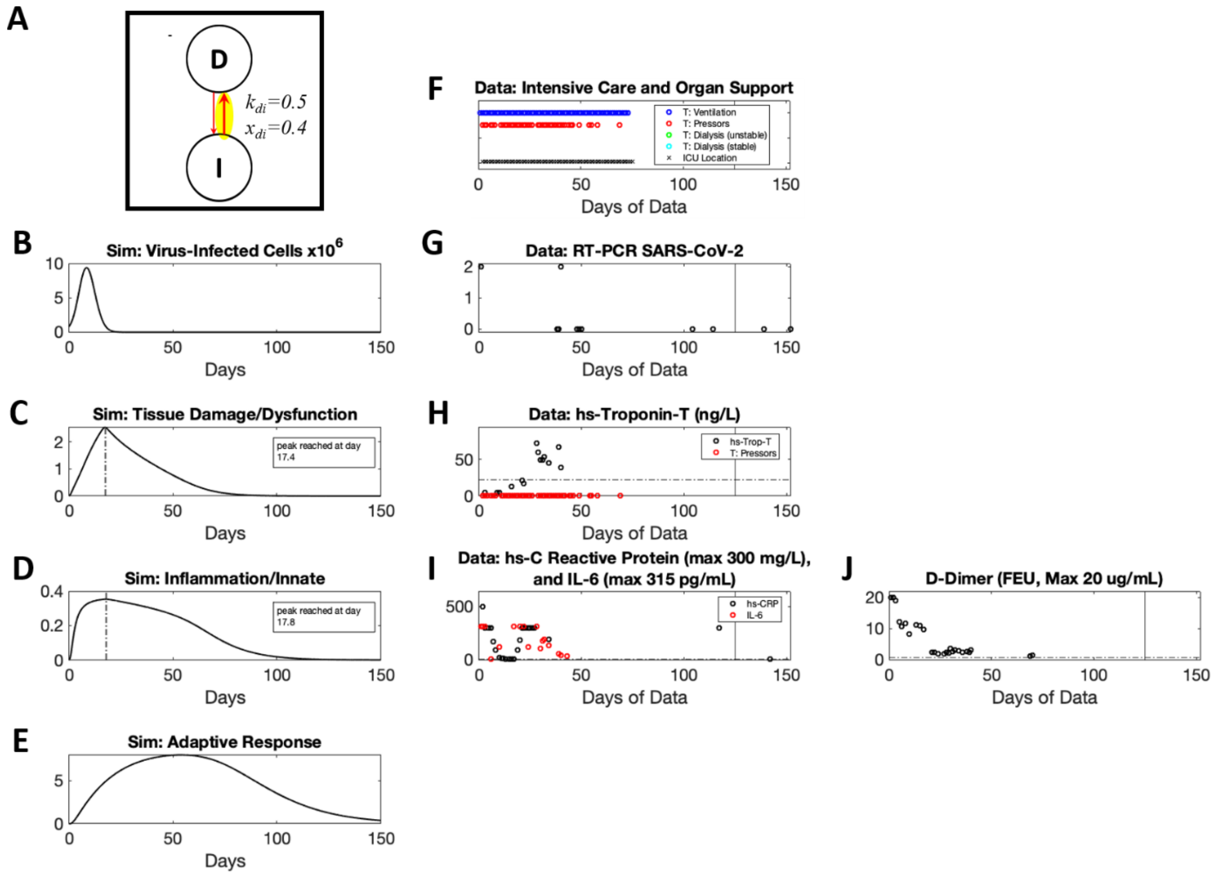

Fig. S2: *Recovery* archetype, Moderate response scenario: simulation vs. patient data. Using modified values of parameters k_{di} and x_{di} compared to Mild scenario as shown in the diagram (**Panel A**), simulated dynamics are shown of C_V (**Panel B**) (Initialized with a C_V =8x10^5 virus-infected cells), D (**Panel C**), I (**Panel D**), and A (**Panel E**). **Panels F-J** show data from a patient in their 40s with no diagnosed medical problems (but elevated hemoglobin A1C suggesting undiagnosed diabetes) who presented after 8 days of fever, cough, and dyspnea at home. They were hypoxic on admission, and despite maximal noninvasive therapy required intubation. The patient was in the intensive care unit for 75 days, during which they were treated for acute respiratory distress syndrome and vasopressor-dependent hypotension. They were given a 5 day course of steroids and IL-6 inhibitor therapy. The patient was liberated from the ventilator by Hospital Day 73 and was able to be discharged to an acute rehabilitation facility on Hospital Day 141.

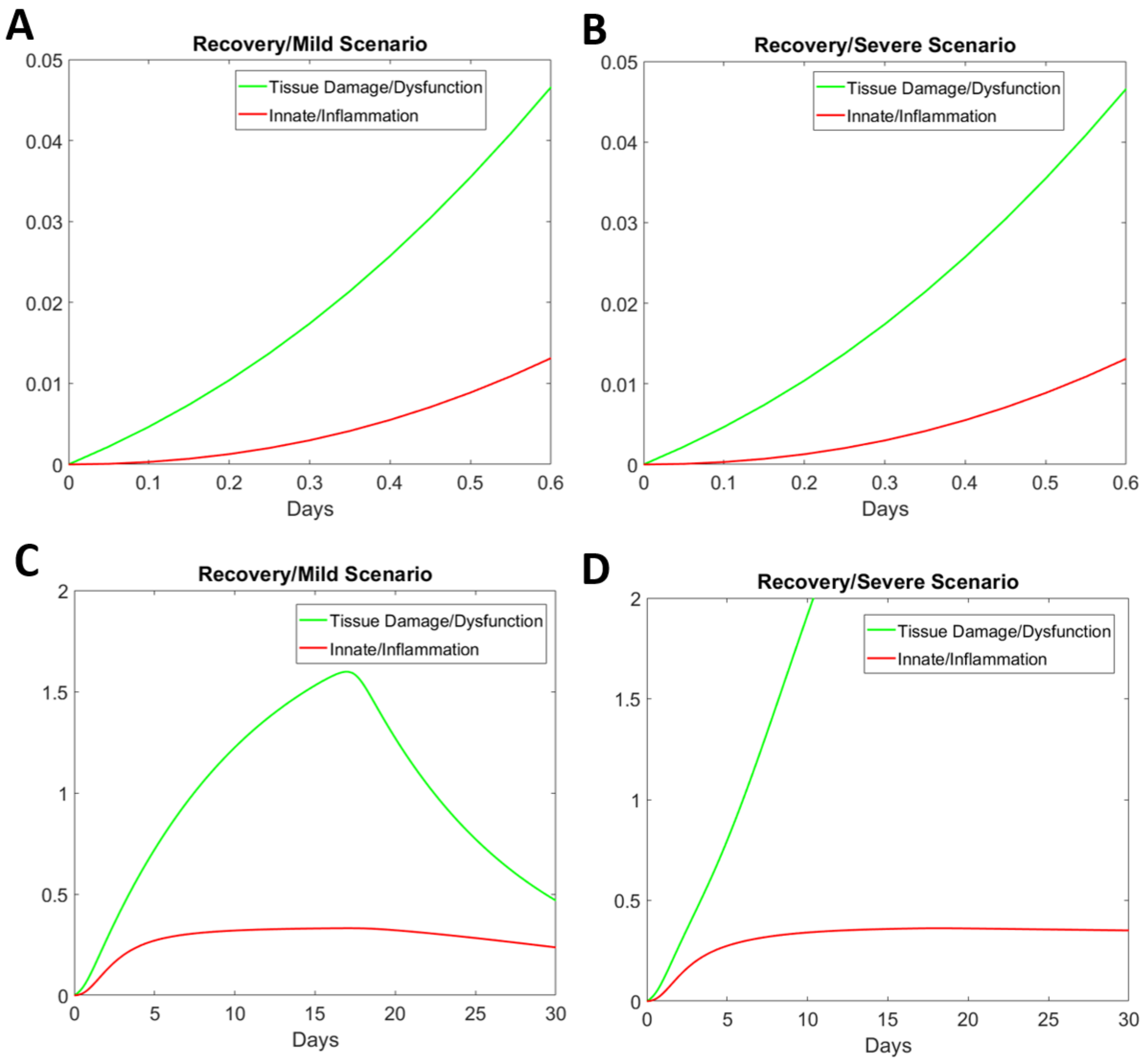

Fig. S3: A magnified view of the time courses for *D* and *I* early in the response in the Mild (Panels A and C) and Severe (Panels B and D) scenarios of the *Recovery* archetype. For the Mild and Severe scenarios, respectively, **Panels A and B** magnify the view of the full time courses of *D* and *I* displayed in **Panels C and D**, showing that initially there is an earlier and much faster rise of *D* relative to *I* in both scenarios (Figs. 3 and 4 in main text). However, this distinction in either scenario is essentially undetectable by Day 1 as seen in **Panel A** and **Panel B**.

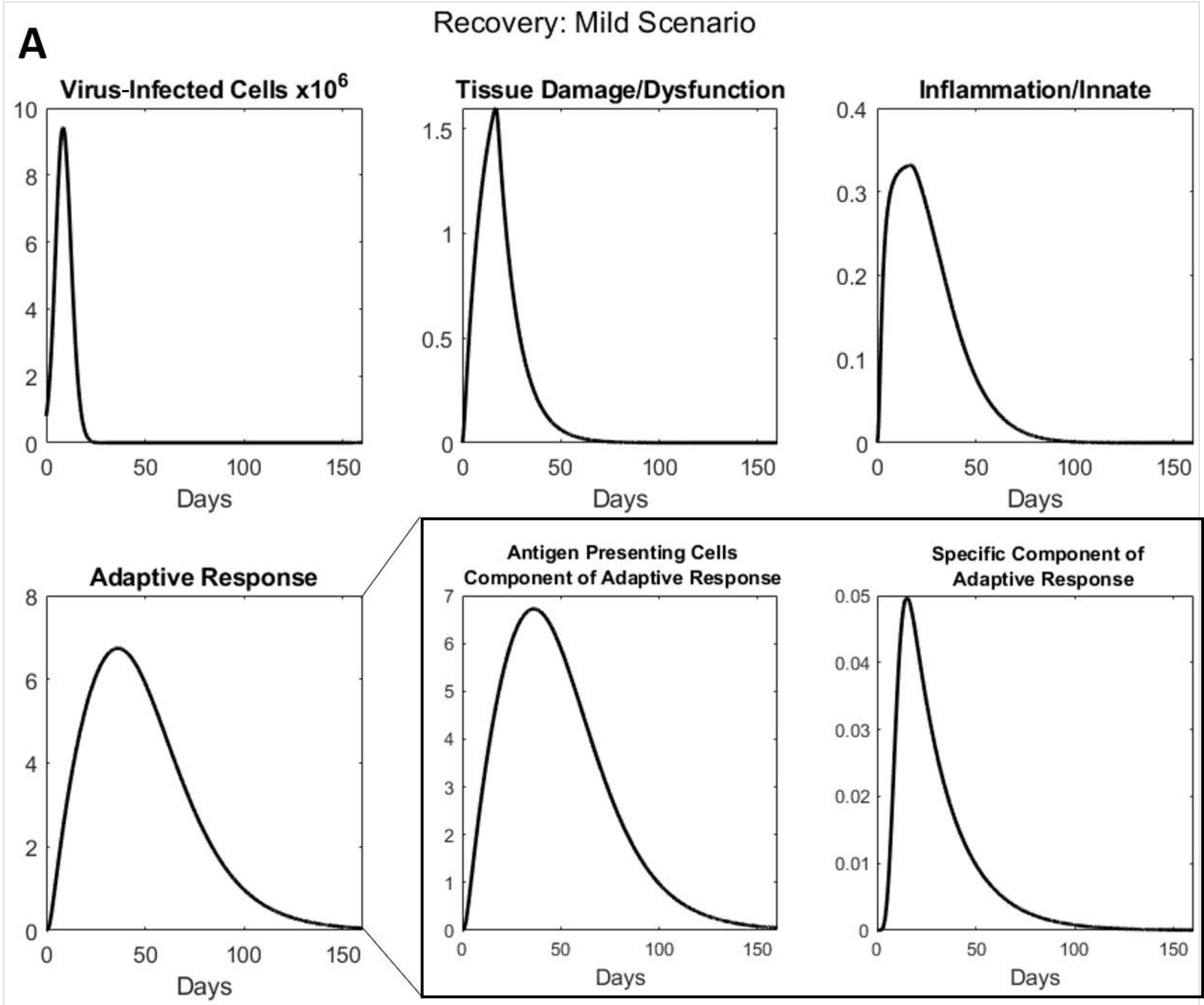

Recovery: Mild Scenario
A
Virus-Infected Cells x10^6
Tissue Damage/Dysfunction
Inflammation/Innate
Adaptive Response
Antigen Presenting Cells Component of Adaptive Response
Specific Component of Adaptive Response
Days

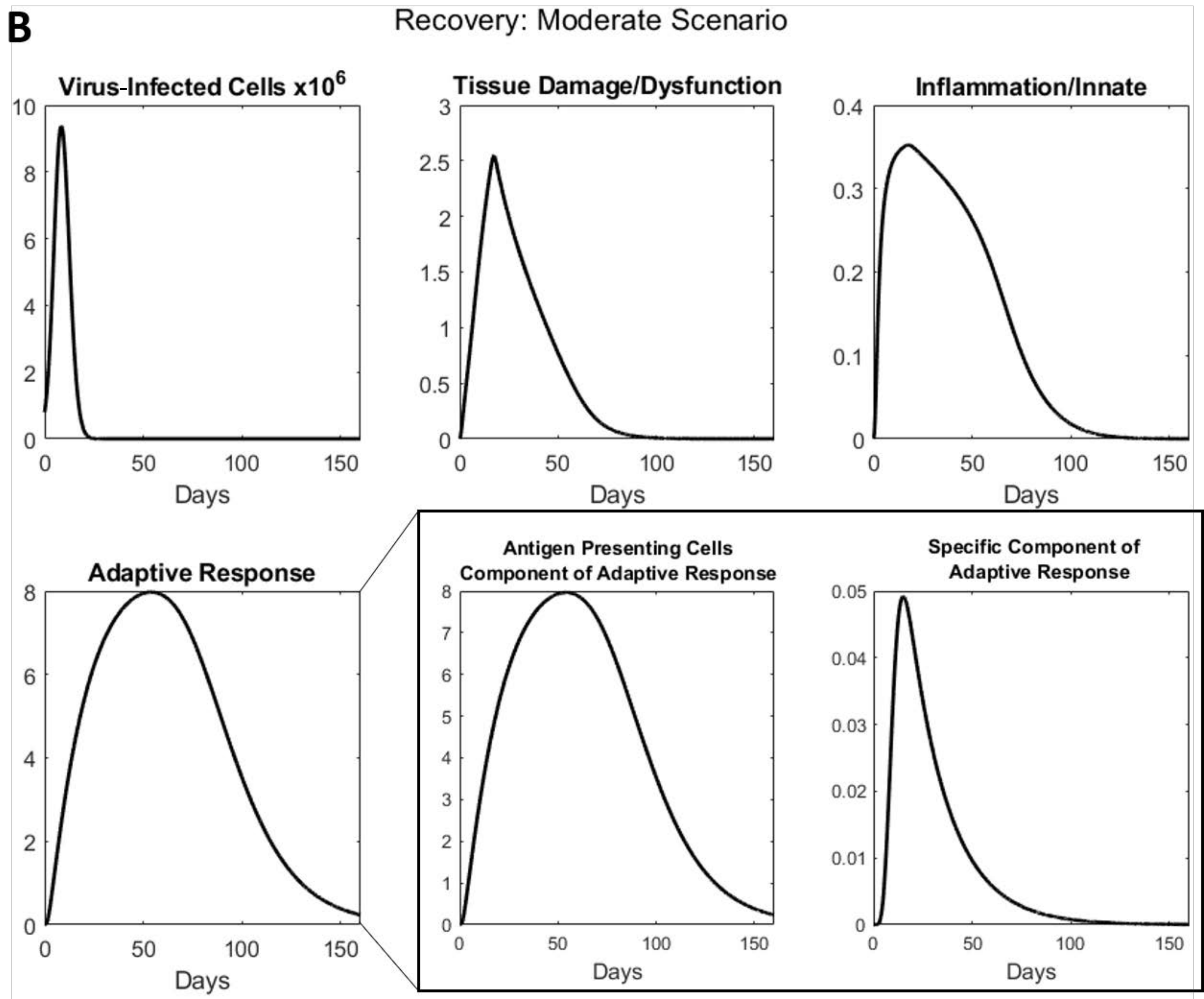
B
Recovery: Moderate Scenario
Virus-Infected Cells x10^6
Tissue Damage/Dysfunction
Inflammation/Innate
Days
Adaptive Response
Antigen Presenting Cells
Component of Adaptive Response
Specific Component of
Adaptive Response

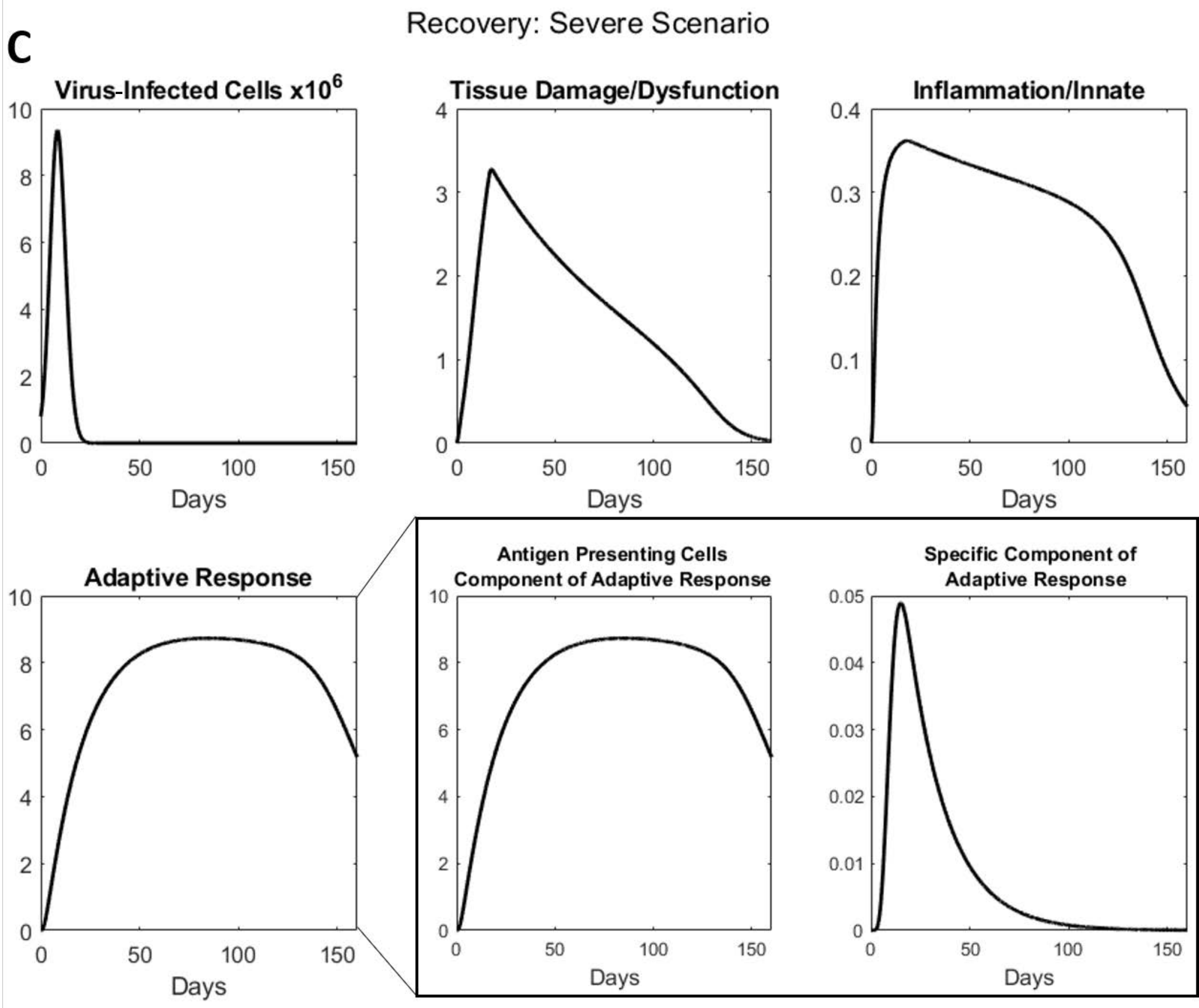

C
Recovery: Severe Scenario
Virus-Infected Cells x10^6
Tissue Damage/Dysfunction
Inflammation/Innate
Adaptive Response
Antigen Presenting Cells
Component of Adaptive Response
Specific Component of
Adaptive Response
Days

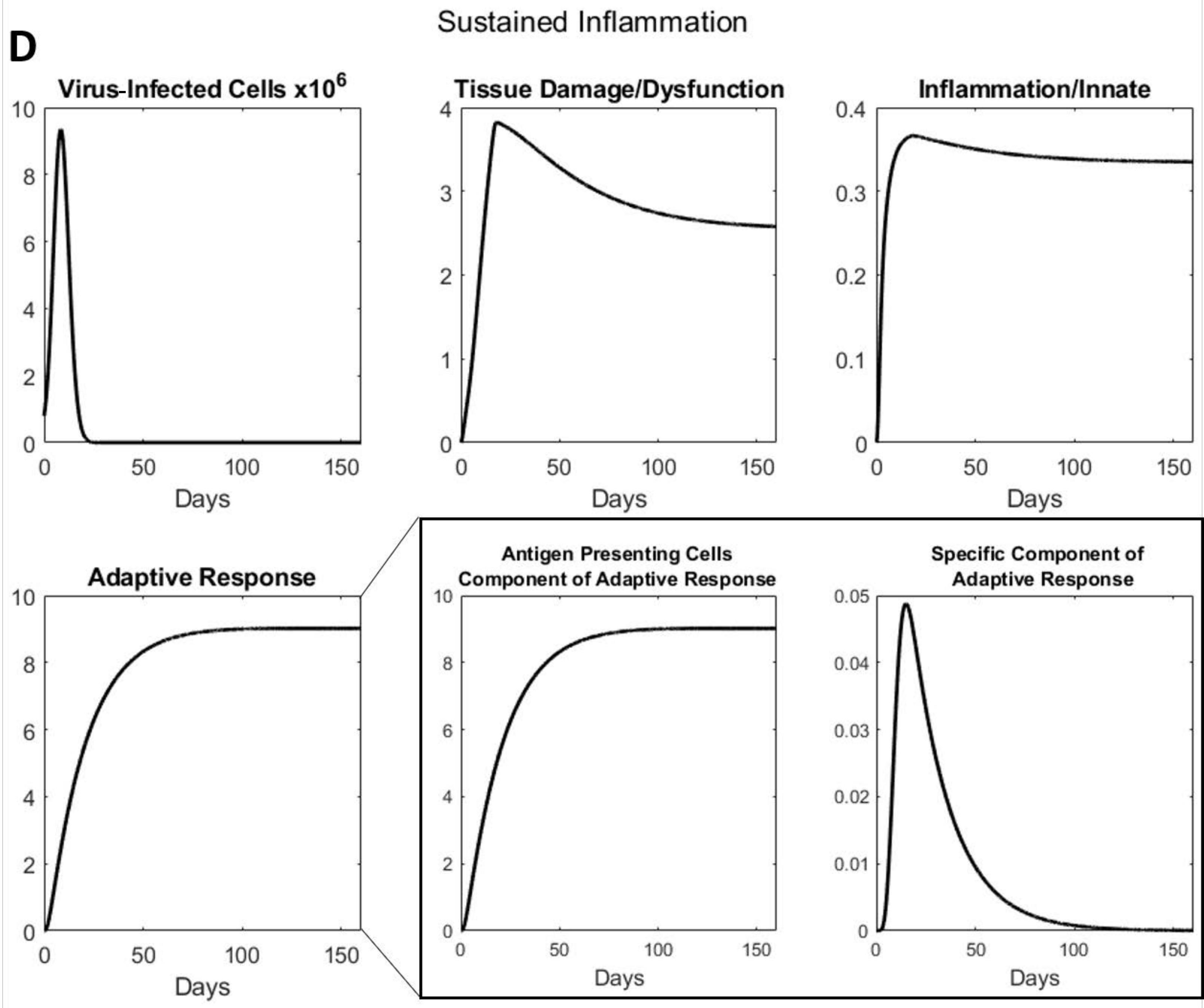

Sustained Inflammation
D
Virus-Infected Cells x10^6
Tissue Damage/Dysfunction
Inflammation/Innate
Adaptive Response
Antigen Presenting Cells
Component of Adaptive Response
Specific Component of
Adaptive Response
Days

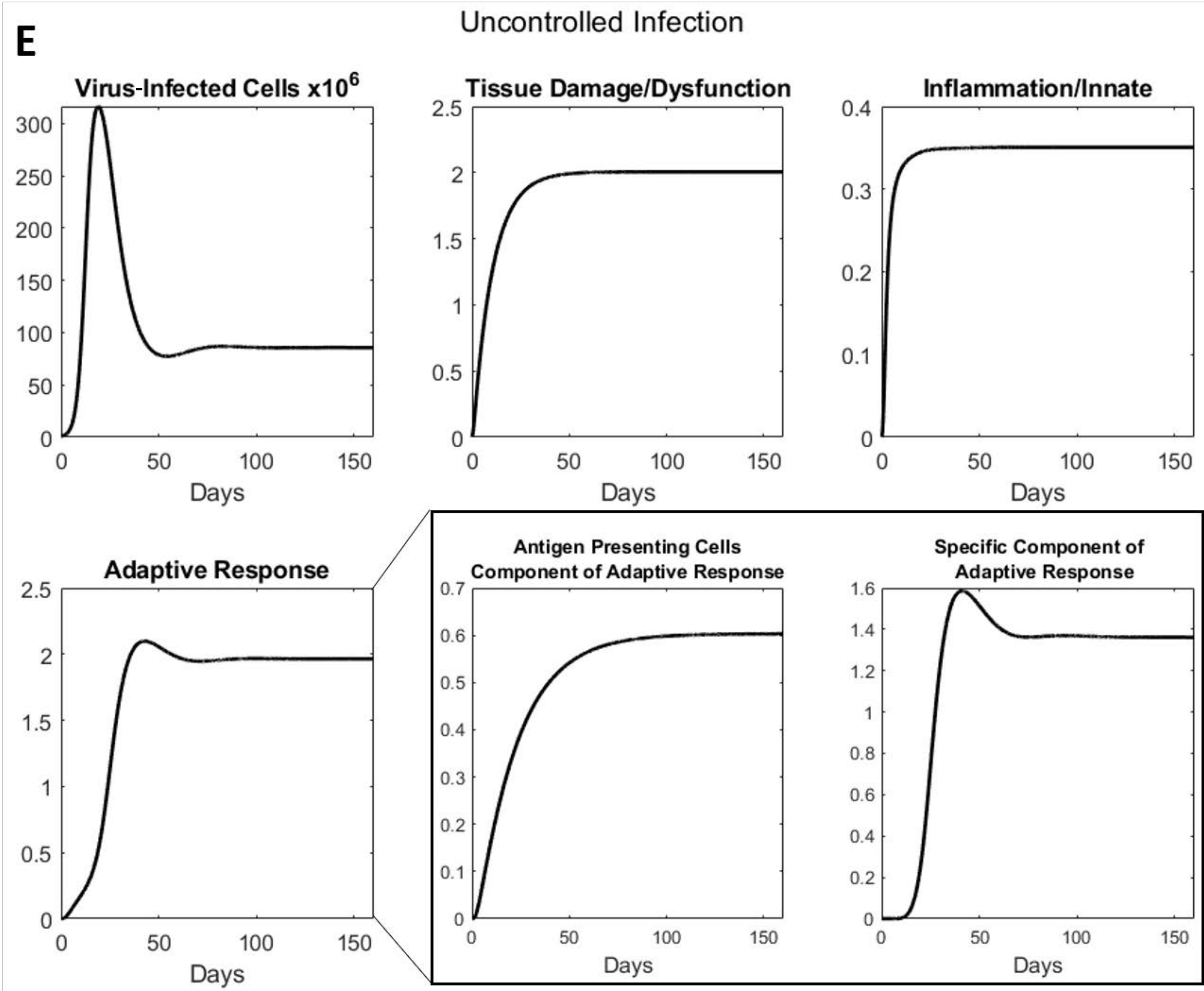
E
Uncontrolled Infection
Virus-Infected Cells x10^6
Tissue Damage/Dysfunction
Inflammation/Innate
Adaptive Response
Antigen Presenting Cells Component of Adaptive Response
Specific Component of Adaptive Response
Days

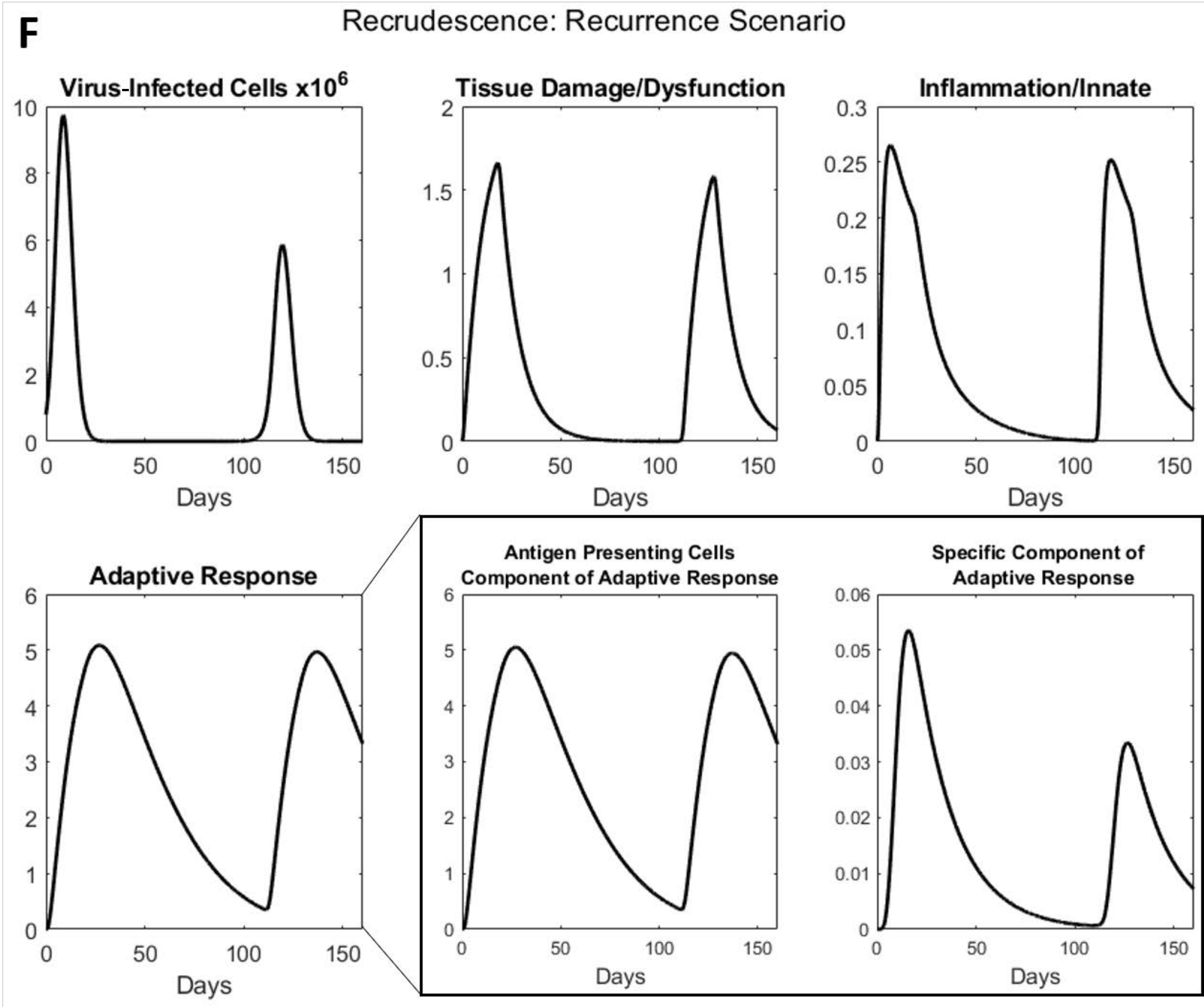
F
Recrudescence: Recurrence Scenario
Virus-Infected Cells x10^6
Tissue Damage/Dysfunction
Inflammation/Innate
Adaptive Response
Antigen Presenting Cells Component of Adaptive Response
Specific Component of Adaptive Response
Days

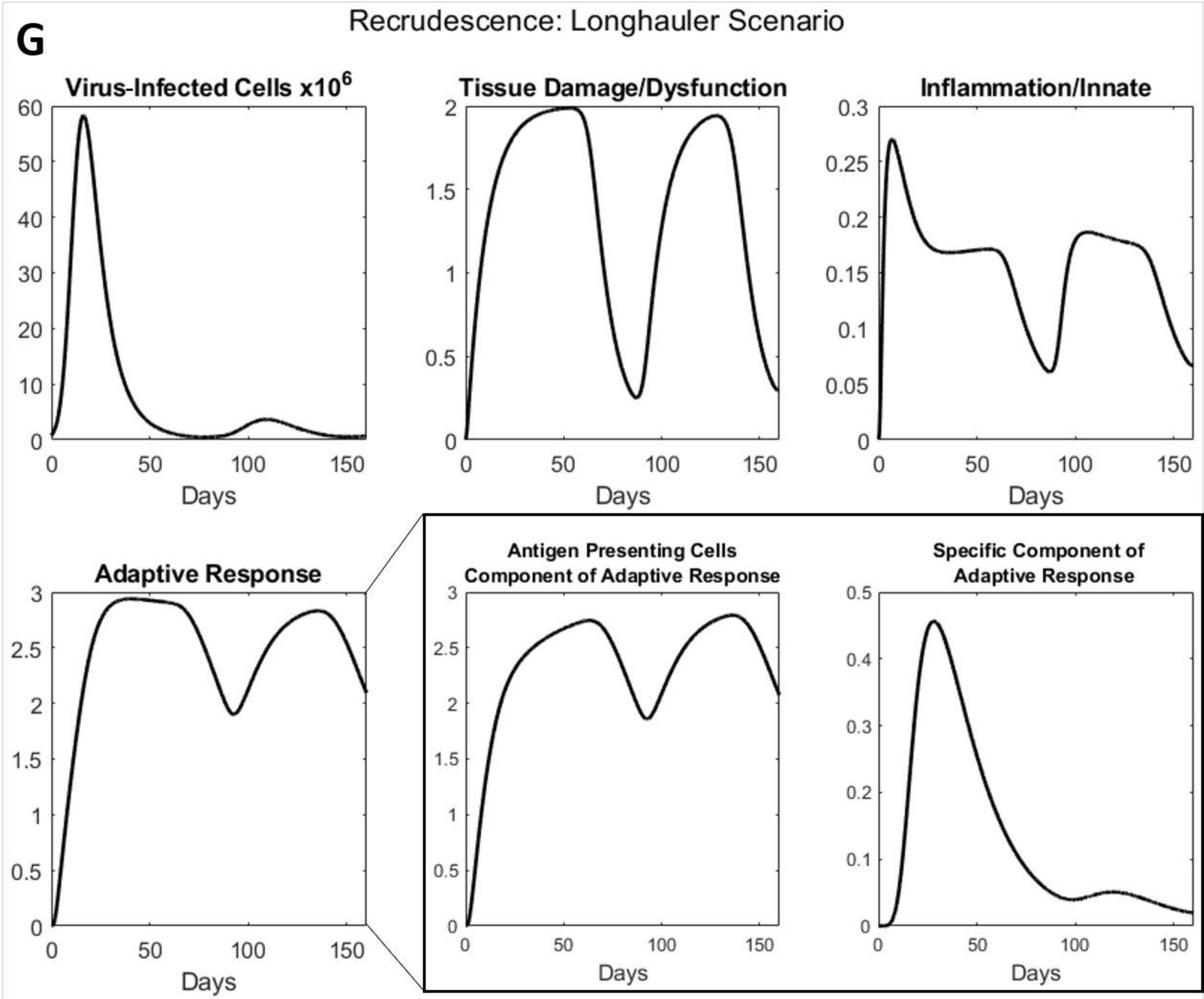

G
Recrudescence: Longhauler Scenario
Virus-Infected Cells x10^6
Tissue Damage/Dysfunction
Inflammation/Innate
Adaptive Response
Antigen Presenting Cells Component of Adaptive Response
Specific Component of Adaptive Response
Days

Fig. S4: Model simulations of core archetype/scenarios with components of the Adaptive Response Variable, *A*. Model simulations for all archetypes/scenarios (**Panels A-G**) shown in Figs. 3, S2, and 4-8, respectively, where a boxed side panel to the right of the Adaptive Response (i.e. *A*) time course for each scenario is included to show the two components of the variable, *A*: the *antigen presenting cells component* (left plot in box) that is primed by *I* to initiate the *specific component* of the adaptive response (right plot in box). The specific component is dependent on the virus-infected cell population, C_V, and will follow along with C_V per the interaction term of *A* with C_V that governs this component; whereas the antigen presenting cells component will be governed by a term dependent on *I* and a source of naïve *A*. Thus, if C_V decays, the specific component will decay as well; otherwise, it will be elevated if C_V is elevated as seen in Figs. S4 **Panels E-G**.

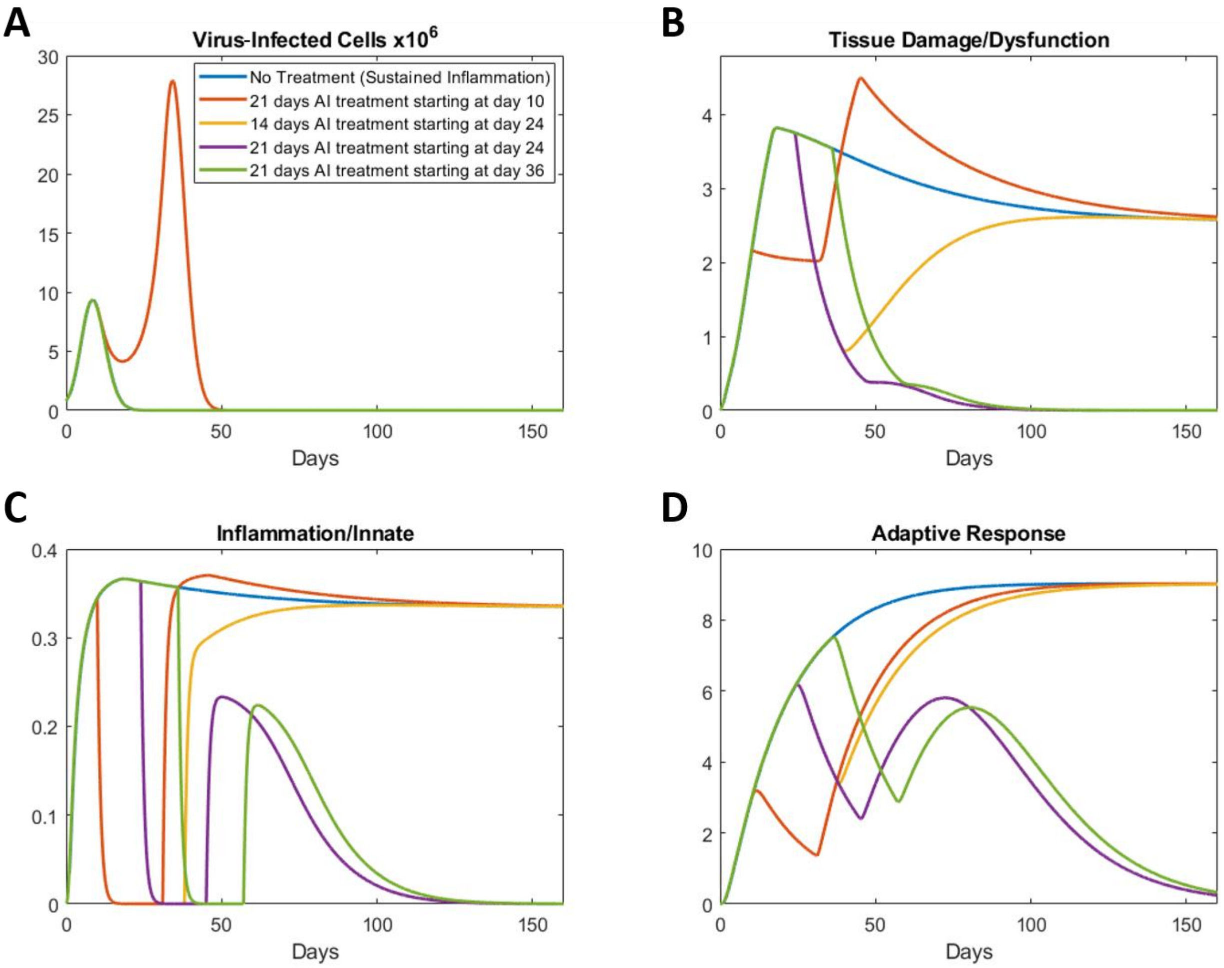

Fig. S5: Various anti-inflammatory (i.e. anti-I) therapy simulations of the *Sustained Inflammation* archetype. Simulated dynamics of C_V (**Panel A**) (Initialized with a C_V =8x10^5 virus-infected cells), D (**Panel B**), I (**Panel C**), and A (**Panel D**) comparing no treatment (blue curve) of the *Sustained Inflammation* archetype which results in a non-recovery outcome to four anti-I treatment simulations of this scenario. Of the four treatment protocols listed in the legend, two were successful to redirect the non-recovery outcome to a recovery outcome; whereas two other treatment protocols converged to the same outcome with no treatment even though there were short-term benefits seen during the treatment period. The timing and duration of the treatment is crucial for a successful outcome.

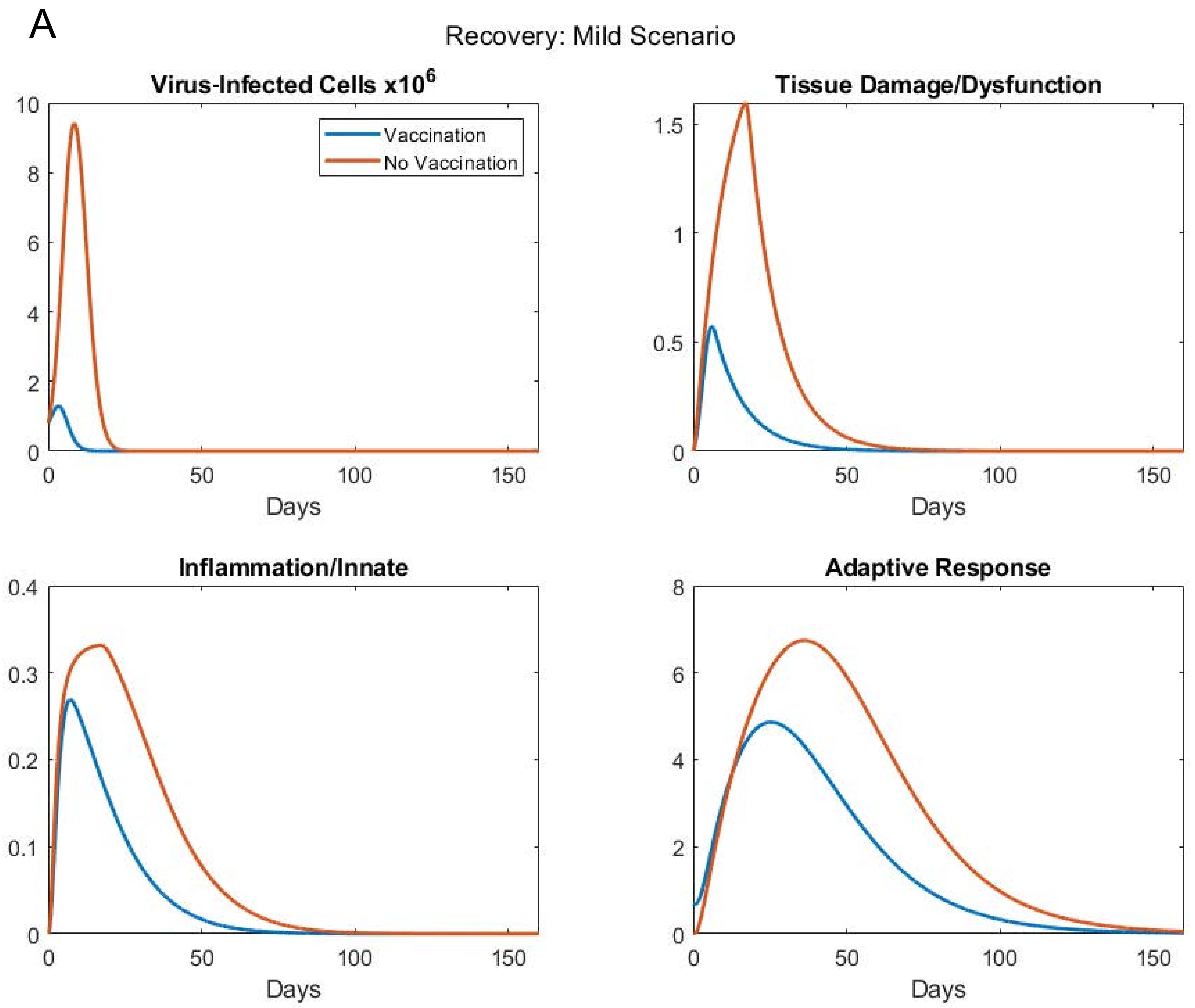
A
Recovery: Mild Scenario
Virus-Infected Cells x10^6
Vaccination
No Vaccination
Tissue Damage/Dysfunction
Inflammation/Innate
Adaptive Response
Days
0
50
100
150
10
8
6
4
2
0
1.5
1
0.5
0.4
0.3
0.2
0.1

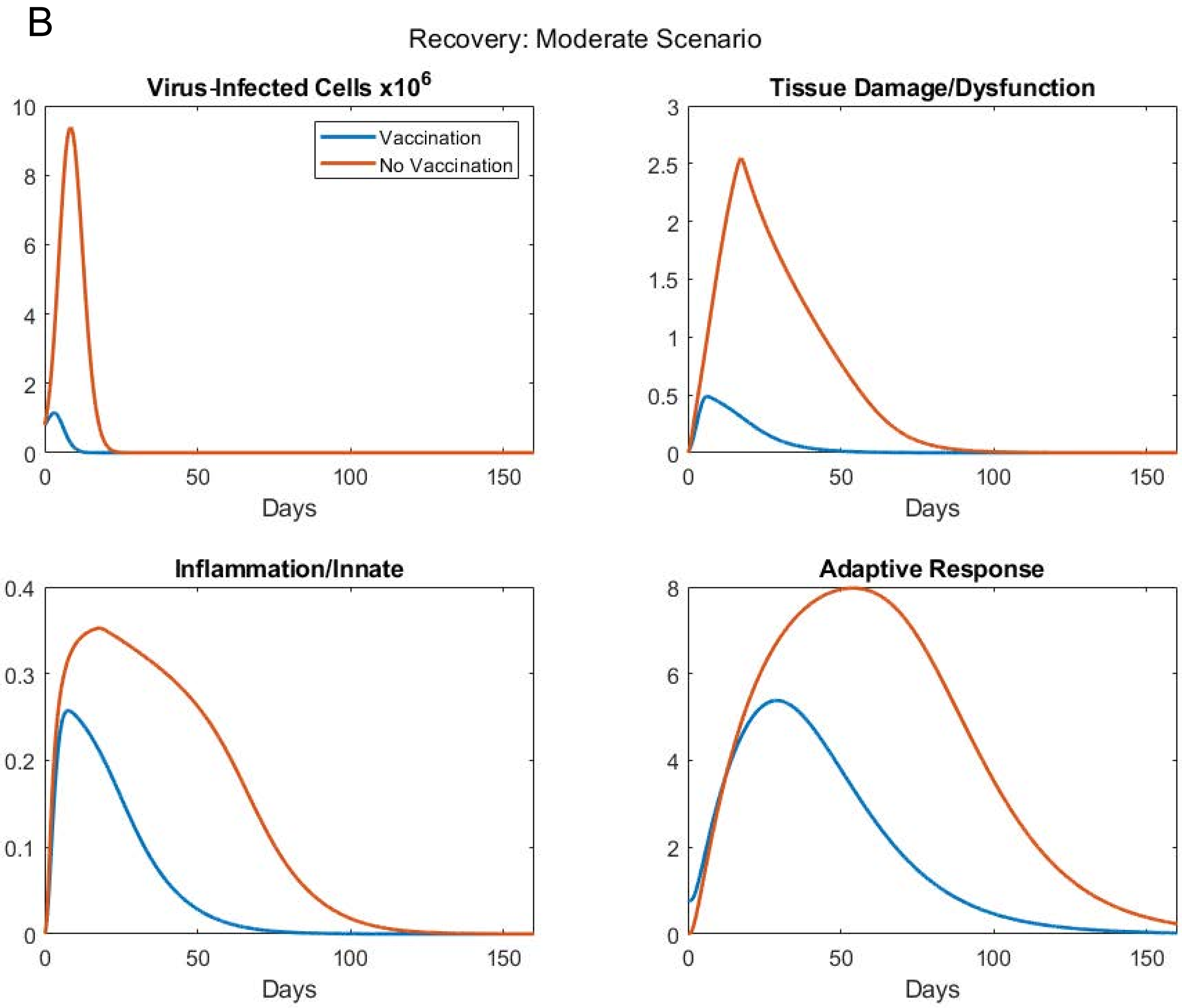
B
Recovery: Moderate Scenario
Virus-Infected Cells x10^6
Vaccination
No Vaccination
Tissue Damage/Dysfunction
Inflammation/Innate
Adaptive Response
Days
Days
Days
Days
0
50
100
150
0
2
4
6
8
10
0.5
1
1.5
2
2.5
3
0.1
0.2
0.3
0.4

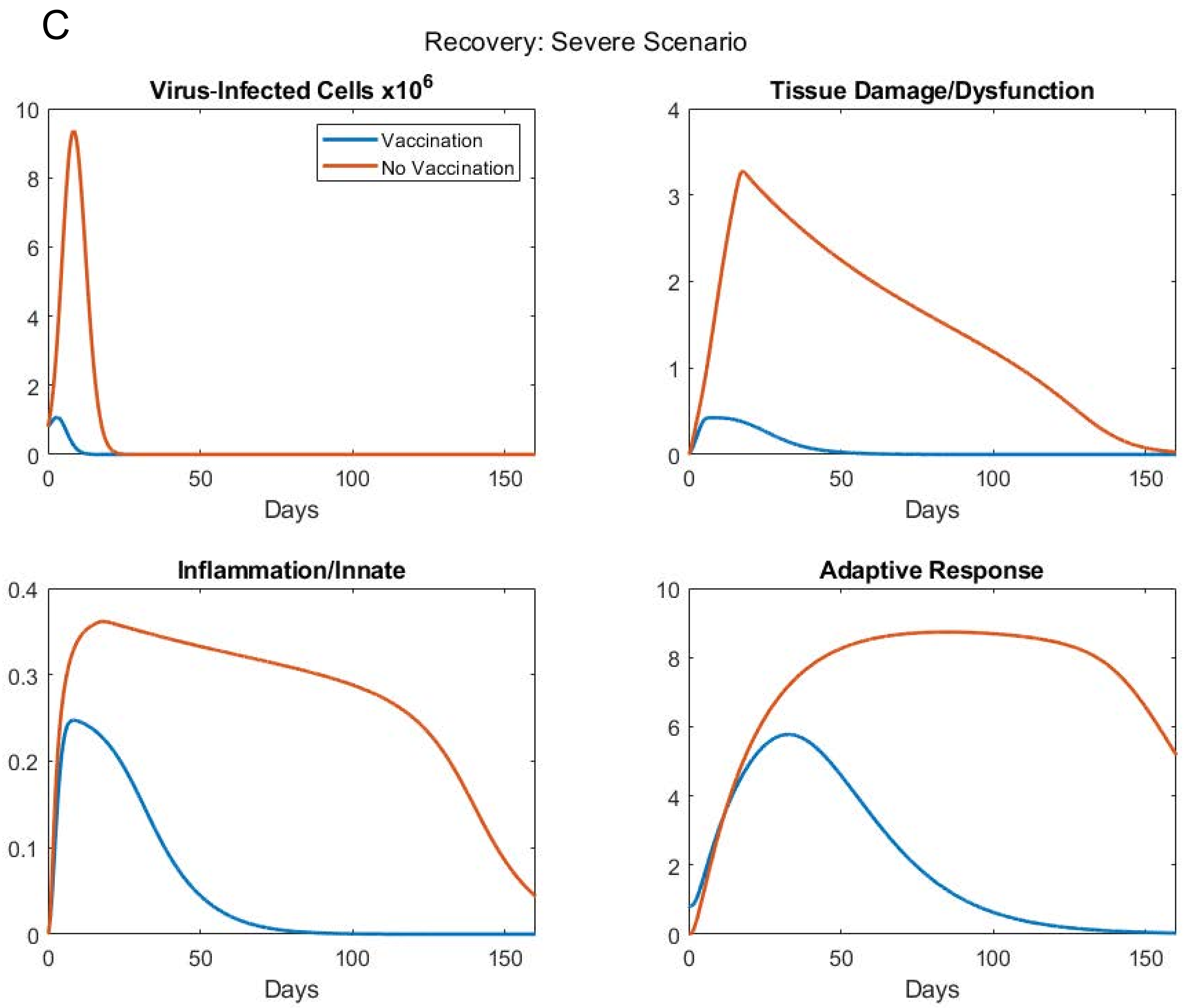
C
Recovery: Severe Scenario
Virus-Infected Cells x10^6
Vaccination
No Vaccination
Tissue Damage/Dysfunction
Inflammation/Innate
Adaptive Response
Days
0
50
100
150
10
8
6
4
2
0
3
1
0.4
0.3
0.2
0.1

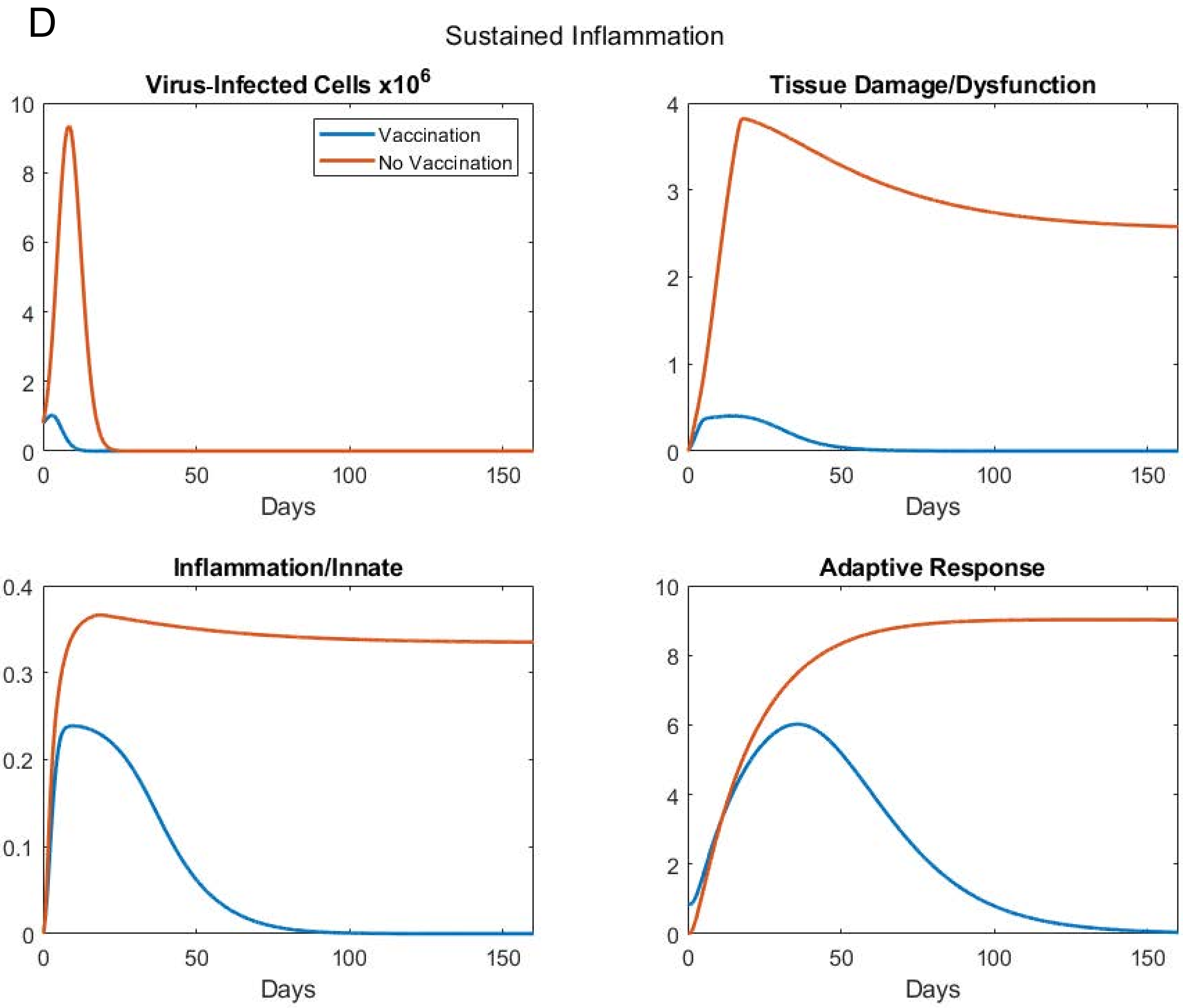
D
Sustained Inflammation
Virus-Infected Cells x10^6
Vaccination
No Vaccination
Tissue Damage/Dysfunction
Inflammation/Innate
Adaptive Response
Days
0
50
100
150
10
8
6
4
2
0
0.4
0.3
0.2
0.1
3
1

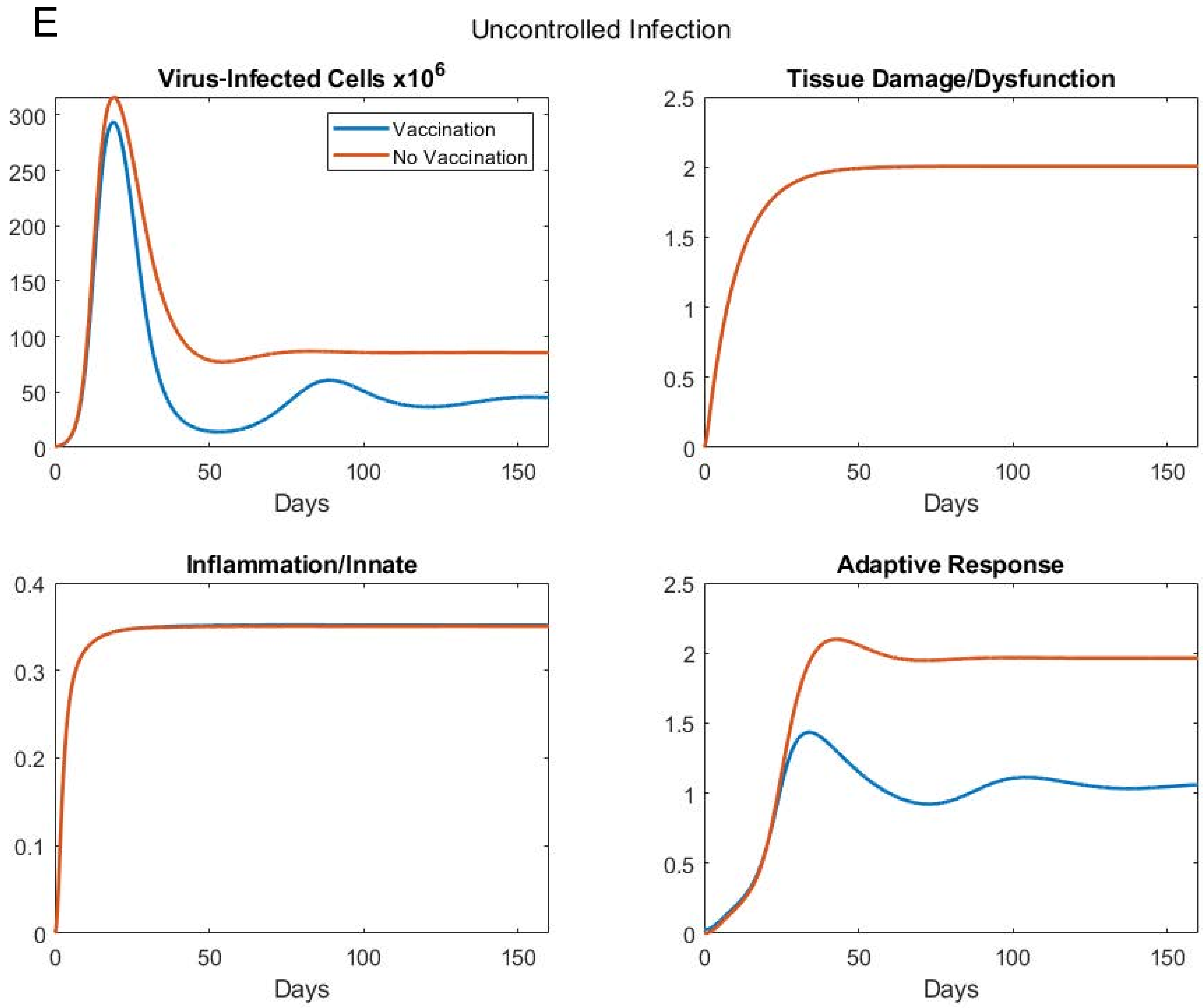

E
Uncontrolled Infection
Virus-Infected Cells x10^6
Vaccination
No Vaccination
300
250
200
150
100
50
0
0
50
100
150
Days
Tissue Damage/Dysfunction
2.5
2
1.5
1
0.5
0
0
50
100
150
Days
Inflammation/Innate
0.4
0.3
0.2
0.1
0
0
50
100
150
Days
Adaptive Response
2.5
2
1.5
1
0.5
0
0
50
100
150
Days

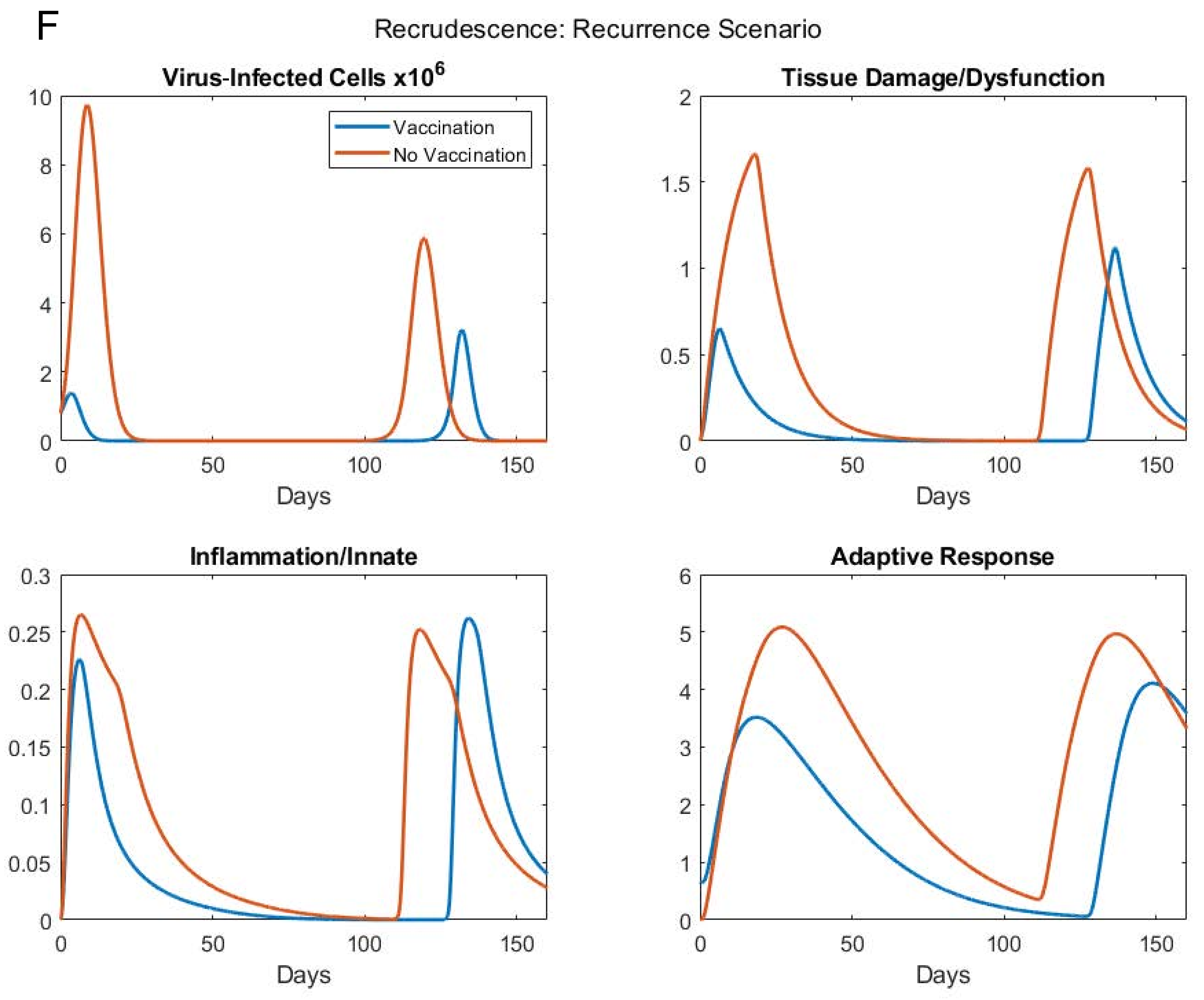
F
Recrudescence: Recurrence Scenario
Virus-Infected Cells x10^6
Vaccination
No Vaccination
Tissue Damage/Dysfunction
Inflammation/Innate
Adaptive Response
Days
0
50
100
150
10
8
6
4
2
1.5
1
0.5
0.3
0.25
0.2
0.15
0.1
0.05
5
3

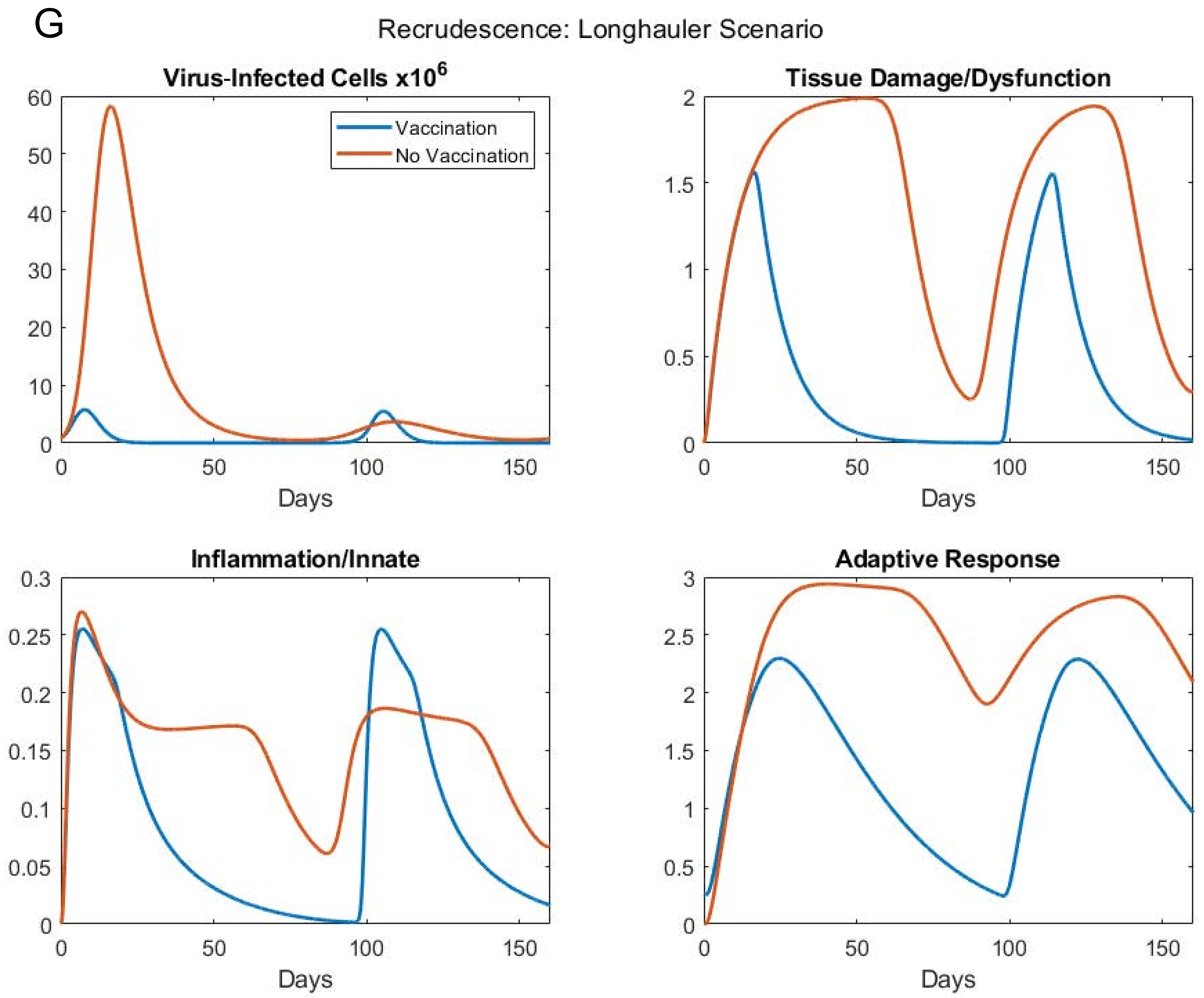
G
Recrudescence: Longhauler Scenario
Virus-Infected Cells x10^6
Vaccination
No Vaccination
60
50
40
30
20
10
0
0
50
100
150
Days
Tissue Damage/Dysfunction
2
1.5
1
0.5
0
0
50
100
150
Days
Inflammation/Innate
0.3
0.25
0.2
0.15
0.1
0.05
0
0
50
100
150
Days
Adaptive Response
3
2.5
2
1.5
1
0.5
0
0
50
100
150
Days

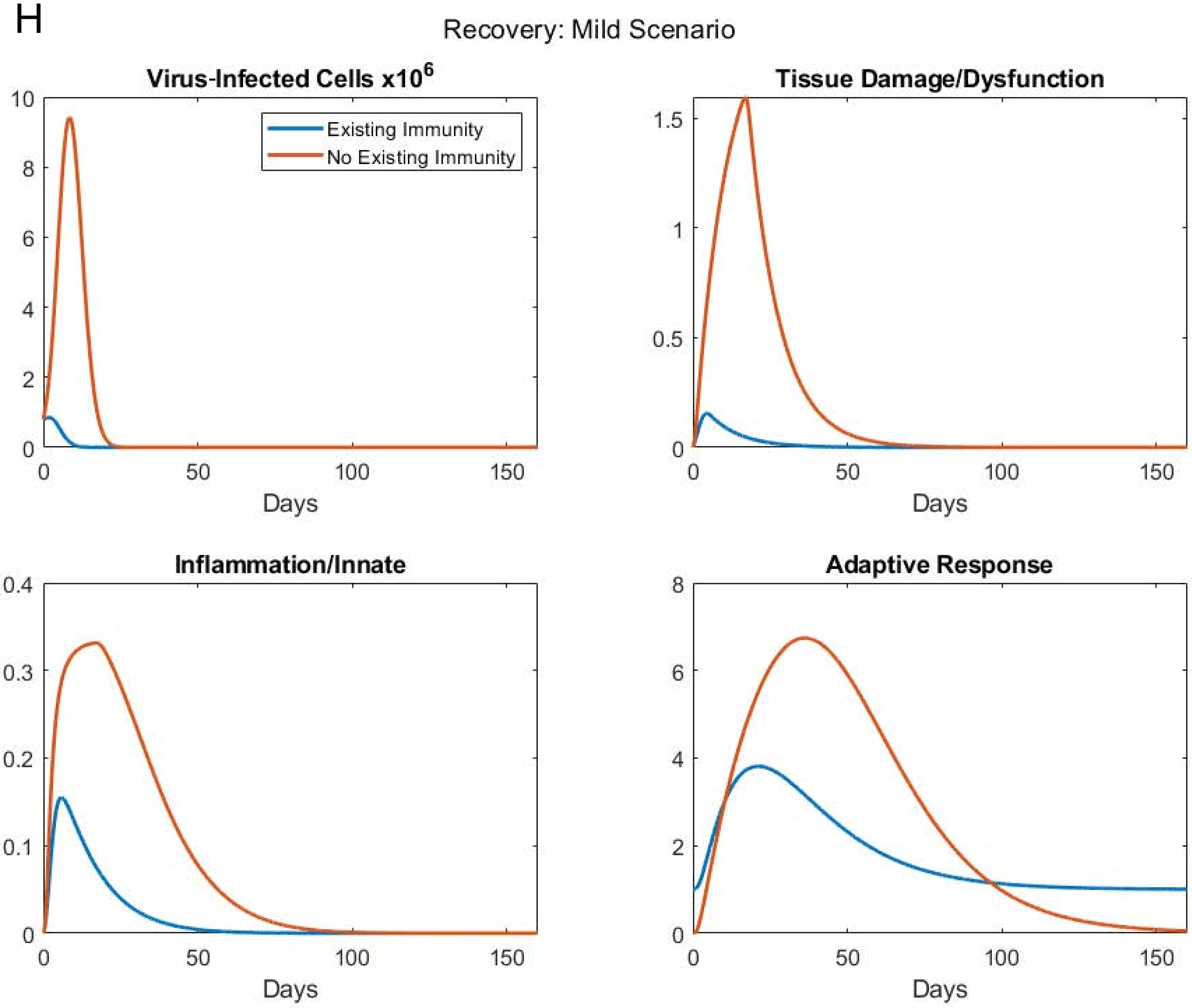
H
Recovery: Mild Scenario
Virus-Infected Cells x10^6
Existing Immunity
No Existing Immunity
10
8
6
4
2
0
0
50
100
150
Days
Tissue Damage/Dysfunction
1.5
1
0.5
0
0
50
100
150
Days
Inflammation/Innate
0.4
0.3
0.2
0.1
0
0
50
100
150
Days
Adaptive Response
8
6
4
2
0
0
50
100
150
Days

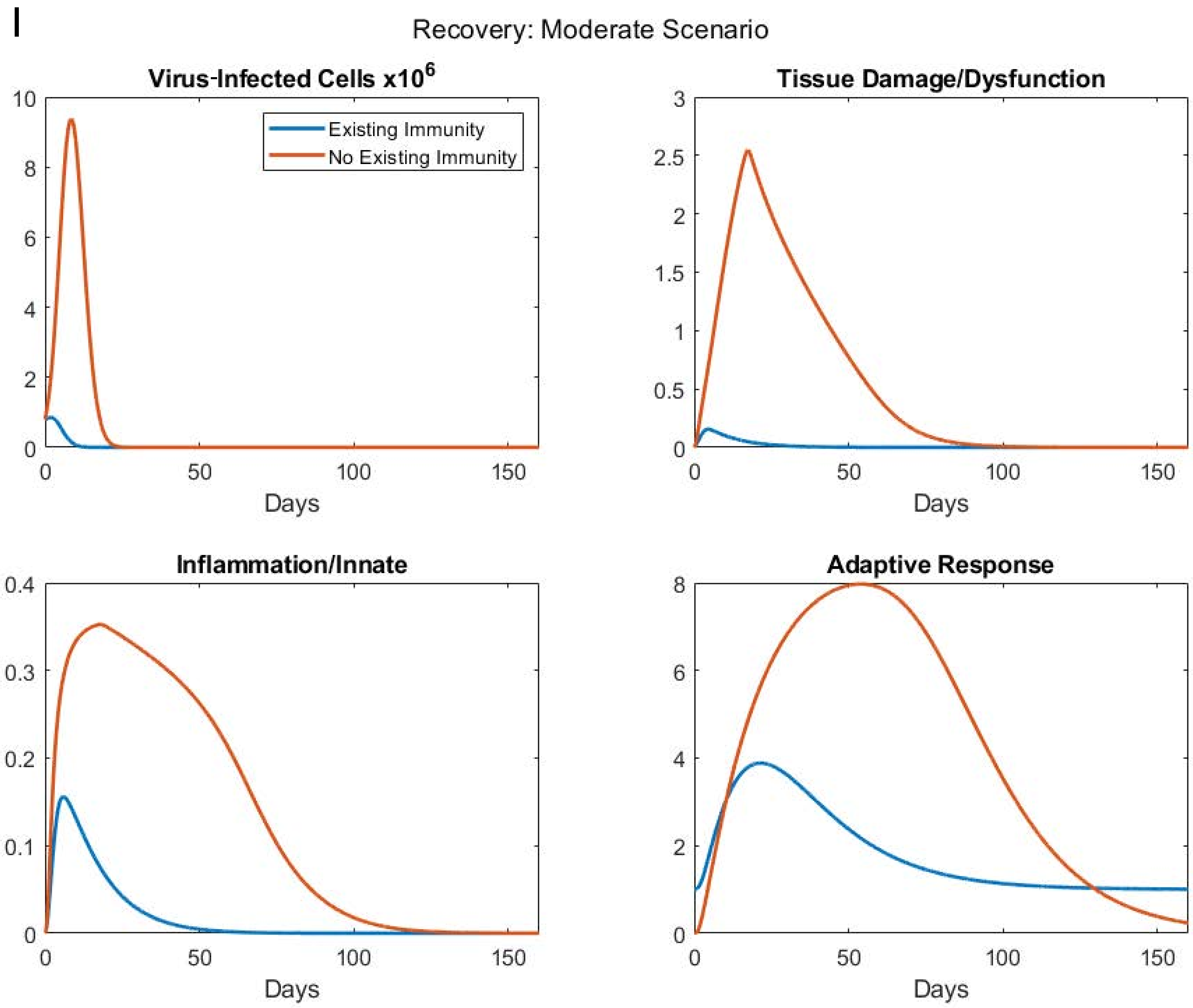
I
Recovery: Moderate Scenario
Virus-Infected Cells x10^6
Existing Immunity
No Existing Immunity
Tissue Damage/Dysfunction
Inflammation/Innate
Adaptive Response
Days
0
50
100
150
10
8
6
4
2
3
2.5
1.5
1
0.5
0.4
0.3
0.2
0.1

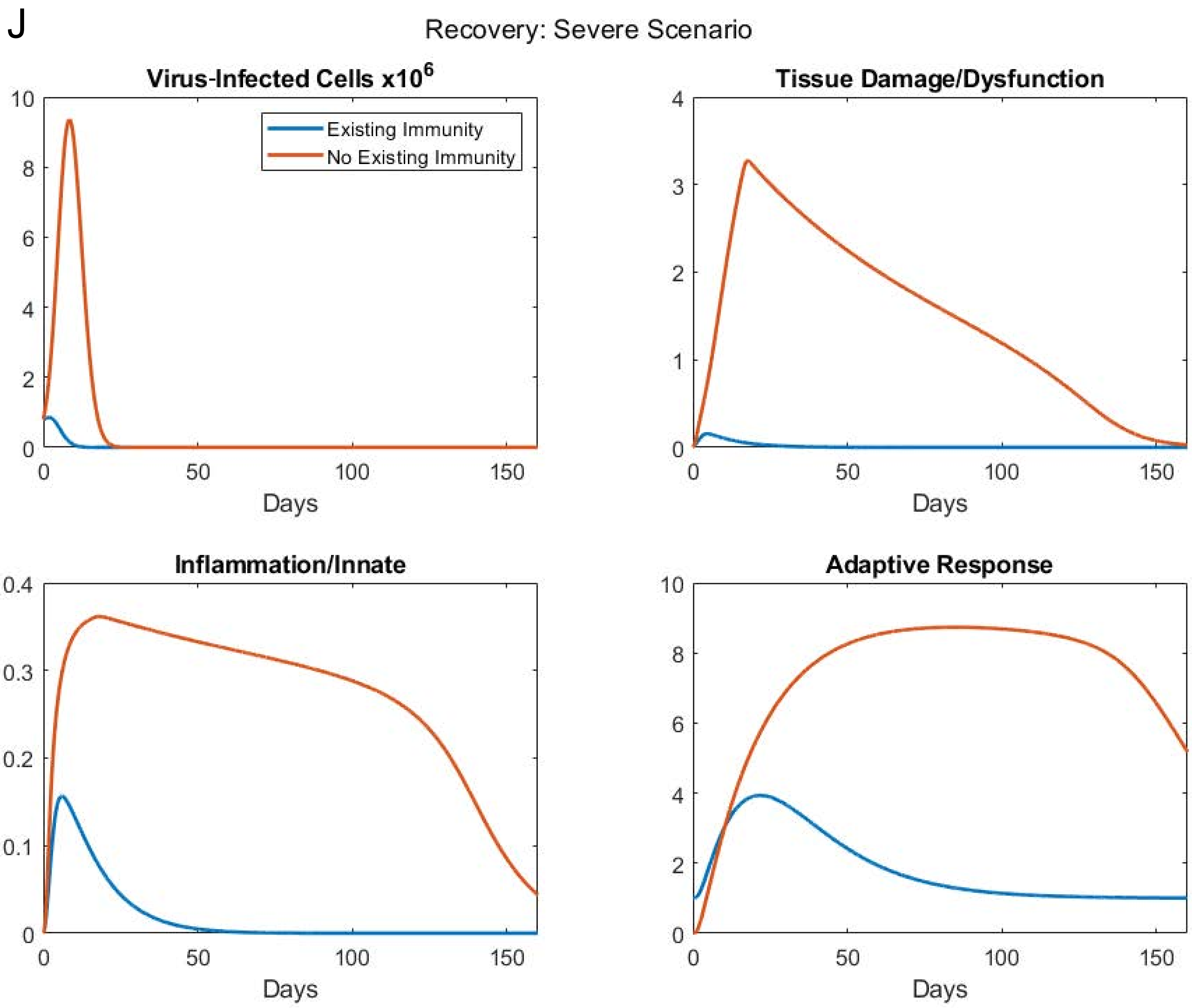
J
Recovery: Severe Scenario
Virus-Infected Cells x10^6
Existing Immunity
No Existing Immunity
10
8
6
4
2
0
0
50
100
150
Days
Tissue Damage/Dysfunction
4
3
2
1
0
0
50
100
150
Days
Inflammation/Innate
0.4
0.3
0.2
0.1
0
0
50
100
150
Days
Adaptive Response
10
8
6
4
2
0
0
50
100
150
Days

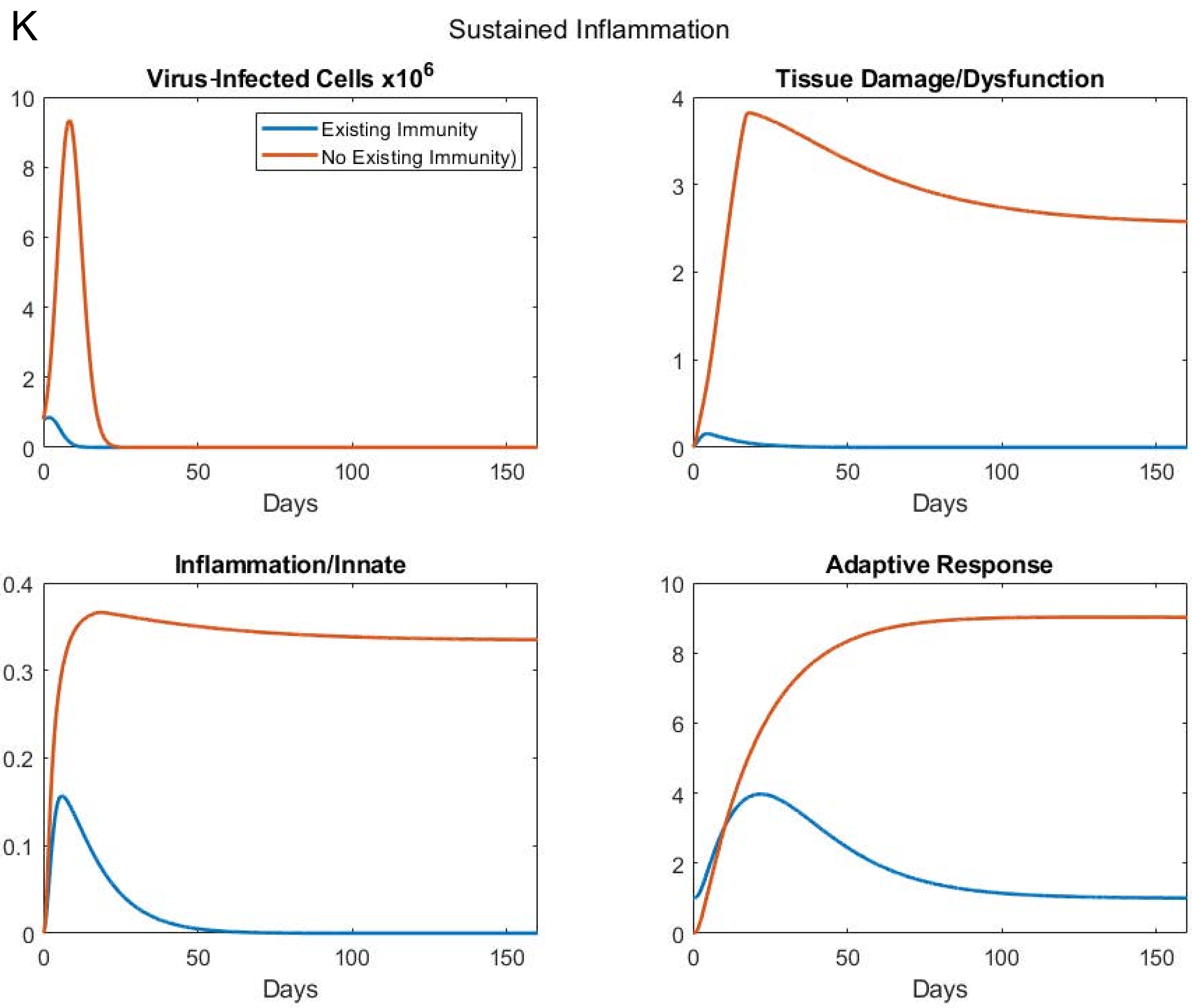
K
Sustained Inflammation
Virus-Infected Cells x10^6
Existing Immunity
No Existing Immunity)
Days
Tissue Damage/Dysfunction
Days
Inflammation/Innate
Days
Adaptive Response
Days

L

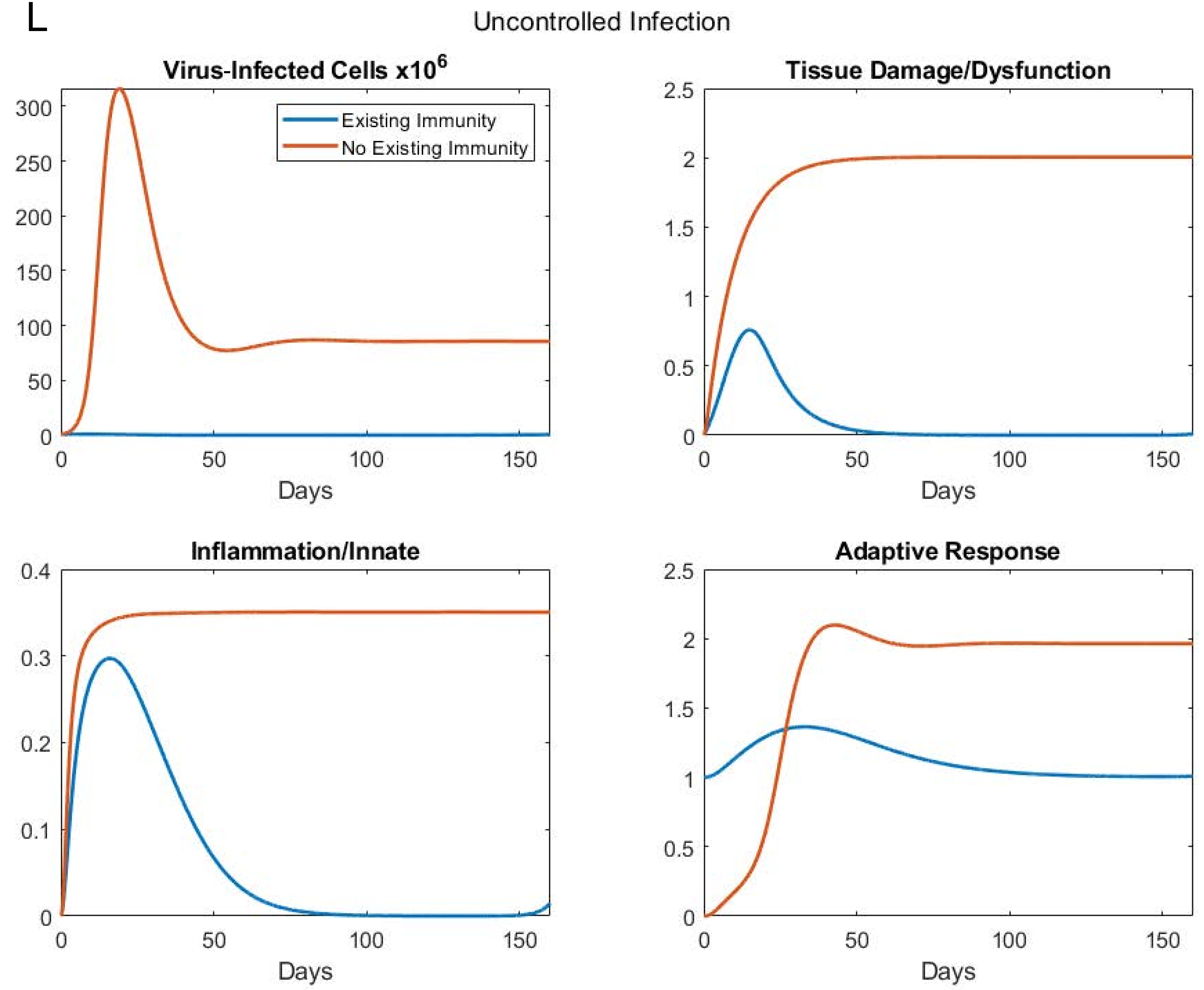
Uncontrolled Infection
Virus-Infected Cells x10^6
Existing Immunity
No Existing Immunity
Days
Tissue Damage/Dysfunction
Days
Inflammation/Innate
Days
Adaptive Response
Days

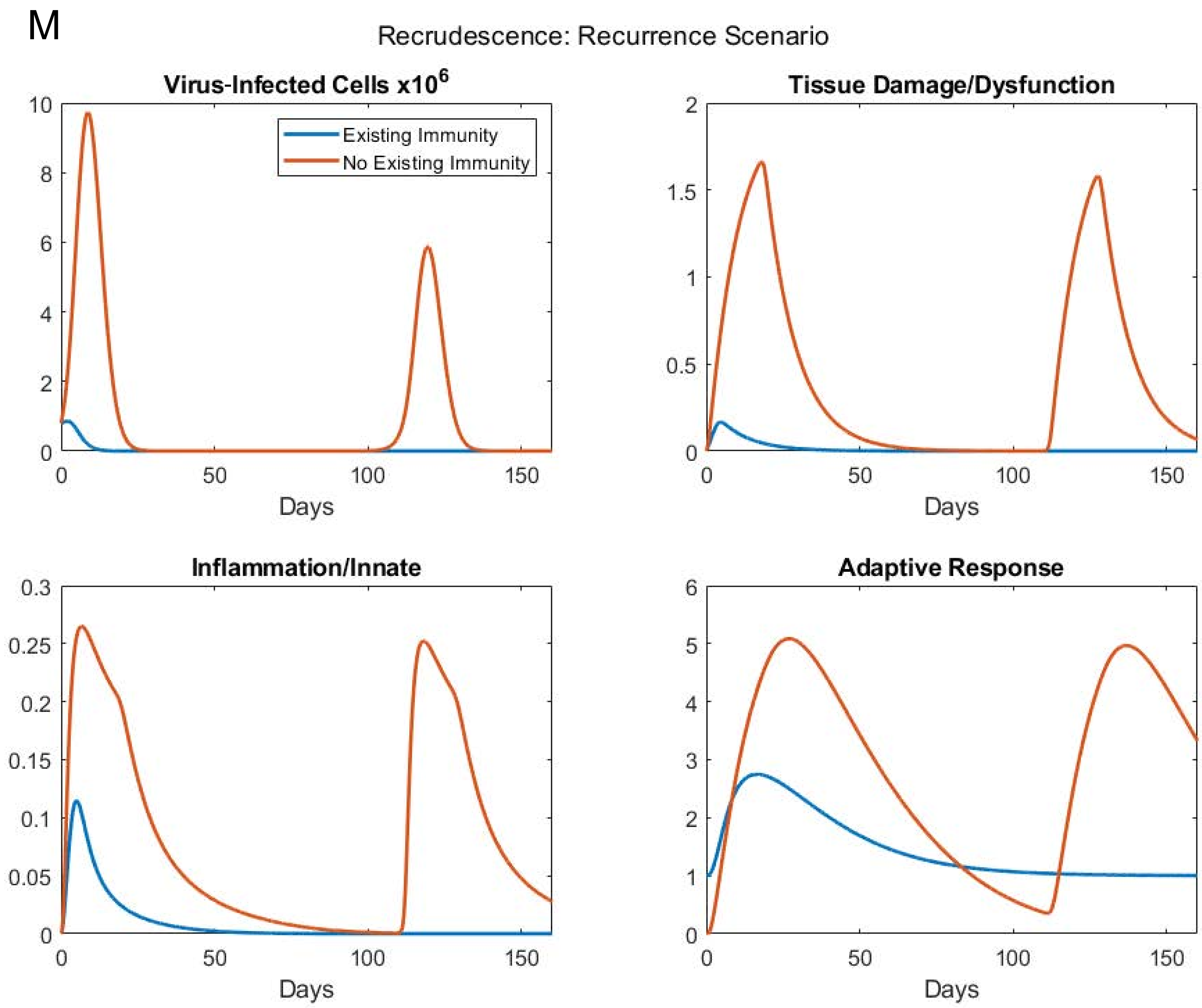
M
Recrudescence: Recurrence Scenario
Virus-Infected Cells x10^6
Existing Immunity
No Existing Immunity
Days
Tissue Damage/Dysfunction
Days
Inflammation/Innate
Days
Adaptive Response
Days

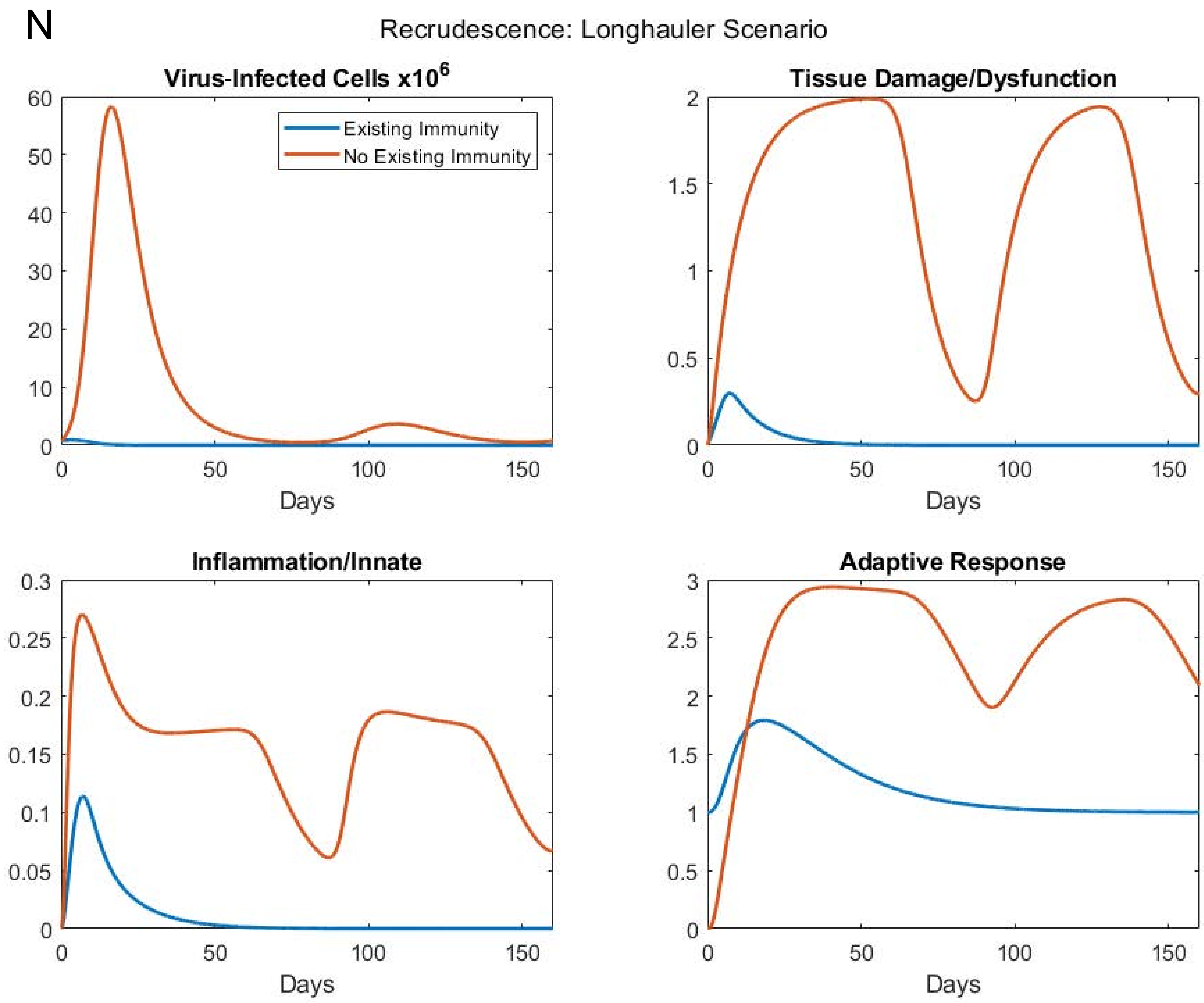
N
Recrudescence: Longhauler Scenario
Virus-Infected Cells x10^6
Existing Immunity
No Existing Immunity
60
50
40
30
20
10
0
0
50
100
150
Days
Tissue Damage/Dysfunction
2
1.5
1
0.5
0
0
50
100
150
Days
Inflammation/Innate
0.3
0.25
0.2
0.15
0.1
0.05
0
0
50
100
150
Days
Adaptive Response
3
2.5
2
1.5
1
0.5
0
0
50
100
150
Days

Fig. S6: Vaccination Simulations with adjuvant-induced short-term immunity (Panels A-G) and with established existing immunity (Panels H-N). Simulated dynamics of C_V (Initialized with C_V =8x10^5 virus-infected cells), D, I, and A under both types of vaccination simulations for Mild (**Panels A and H**), Moderate (**Panels B and I**), and Severe (**Panels C and J**) scenarios of the *Recovery* archetype; *Sustained Inflammation* archetype (**Panels D and K**); *Uncontrolled Infection* archetype (**Panels E and L**); and the Recurrence (**Panels F and M**) and Longhauler (**Panels G and N**) scenarios of the *Recrudescence* archetype. Under the short-term vaccination protocol (**Panels A-G**), all but two scenario outcomes remain unchanged: The Sustained Inflammation and Recurrence scenarios are converted to a Recovery type. Under the Existing Immunity simulations **(Panels H-N)**, all scenarios remain or are converted to a Recovery type.
