# Supplementary material for: Divergent COVID-19 Disease Trajectories Predicted by a DAMP-Centered Immune Network Model
Source: Front Immunol. 2021 Oct 28;12:754127. doi: 10.3389/fimmu.2021.754127 (PMC8582279; doi:10.3389/fimmu.2021.754127)
Supplement: Supplementary file 2 [file DataSheet_2.pdf]

SUPPLEMENTARY MATERIALS AND METHODS

Overview of mathematical model of the immune-inflammatory response to SARS-CoV-2.

Figure 1 of the main text provides a schematic of the interactions and processes considered for our mathematical model, which represents an abstraction and simplification of the myriad of immune events occurring during a viral infection. It considers the interaction of four compartments: 1) Virus-infected cells, C_V, 2) Tissue/organ/organism dysfunction that drives inflammation and serves as an indicator of host health, D, 3) Innate inflammatory components, I, and 4) Adaptive Immune components including antigen presenting cells, CD8 T cells, and virus-specific antibodies, A. The mean field dynamics are described by differential equations (1)-(4). Each compartment represents a variety of mediators sharing a common role in the response.

$\frac{dC_V}{dt} = k_{vg}C_V(1 - k_{vs}C_V)f_1(1, x_{vainv}A) - k_{va}A \cdot C_V$	(1)
$\frac{dD}{dt} = k_{dv}f_2(C_V, x_{dv}) + k_{di}f_2(I, x_{di}) - \mu_d D$	(2)
$\frac{dI}{dt} = s_{ir}\frac{R}{\mu_{ir} + R} - \mu_i I$	(3)
$\frac{dA}{dt} = \frac{k_{ai} \cdot s_{a0}}{\mu_{a0} + k_{ai}I} \cdot I \cdot \frac{x_{1ai}^2}{x_{1ai}^2 + I^2} + k_{ag} \cdot C_V \cdot \frac{A^2}{x_{ag}^2 + A^2} - \mu_a A + A_e$ $= k_{ai}A_0 \cdot I \cdot \frac{x_{1ai}^2}{x_{1ai}^2 + I^2} + k_{ag} \cdot C_V \cdot \frac{A^2}{x_{ag}^2 + A^2} - \mu_a A + A_e$	(4)

where $f_1(x, y) = \frac{\mathrm{x}}{1+y^2}$ is used in equation (1) and only when vaccination simulations are implemented; and where $f_2(x, y) = \frac{x^6}{y^6+x^6}$ is used in equation (2). The R equation used in equation (3) is given by

$R = (k_{id}D + k_{ii}I)f_1(1, x_{iainv}A) = \frac{k_{id}D + k_{ii}I}{1 + (x_{iainv}A)^2}$	(5)

The virus-infected cell population, C_V, is scaled to have units of 10^6 cells, such that a value of $V = 10^{-6}$ corresponds to one infected cell. The variables *I* and *A* have arbitrary units given that they each represent various cell types, cytokines, chemokines, and other mediators considered to be a part of the respective response. The units of *D* are also arbitrary as it is not linked to one specific biomarker. Parameters are described in Table S1, some of which are based on our previous work (*37, 91*).

Virus-infected Cells, C_V

Viral-cell infection dynamics are often modeled in terms of compartments for the free/extracellular viral particles, target or susceptible cells, viral infected cells, and sometimes 'unproductive' viral infected cells (*33-36*). For example, in Zarnitsyna et al. (*36*), a model was considered for influenza included viral particles, *P*, susceptible cells *S*, and viral-infected cells C_V:

$$\frac{dS}{dt} = -k_{SP}\, SP - k_S S$$

$$\frac{dP}{dt} = k_V C_V - k_P P$$

$$\frac{dC_V}{dt} = k_{SP}\, SP - k_V C_V$$

where the C_V, *P* and *S* are fractions and the decay and interaction rates can depend on other compartments. We reduced these dynamics to a single compartment by assuming 1) viral particle clearance from the vicinity of cells is fast compared to their transmission rate between cells and thus the viral dynamics can be approximated to be in quasi-steady state, $P = (k_V/k_P)C_V$; and 2) that the intrinsic death rate of susceptible cells is slow compared to the time course of the infection, giving $S + C_V = 1$ (i.e. $S + C_V$ is conserved) and thus we can set $S = 1 - C_V$. This results in a single compartment reduced dynamics for the virus-infected cells C_V given as:

$$\frac{dC_V}{dt} = \tilde{k} C_V (1 - C_V) - k_V C_V$$

We use this as the basis for our C_V compartment for the virus-infected cell population that increases as a result of the process of the virus hijacking the cellular machinery of its target cell to produce virions intracellularly that are released into the extracellular environment to infect new target cells. This construct along with our scaling choice implies that time zero in our model corresponds to the time when there are $\mathrm{C}_V \times 10^6$ infected cells already present. We model this increase of the virus-infected cell population, C_V, with the density dependent logistic growth term: $k_{vg}\mathrm{C}_V(1 - k_{vs}\mathrm{C}_V)$. Logistic growth is bounded above, which for this population can be best attributed to a limited number of target cells

for the virus to infect (*33*). This type of growth starts off exponential and then slows as the population nears a carrying capacity, $\frac{1}{k_{vs}}$.

The adaptive response, A, has dual action on the virus-infected cell population. A inhibits the growth of C_V via virus-specific antibodies by preventing additional target cells from becoming infected. This effect is modeled by dividing the growth term of C_V by $1 + (x_{vainv}A)^2$:

$$\frac{k_{vg}C_V(1 - k_{vs}C_V)}{1 + (x_{vainv}A)^2}$$

However, this mechanism of inhibition is only used when considering vaccination simulations (including simulations of existing SARS-CoV-2-specific immunity). If $x_{vainv} \ll 1$ then inhibition is weak until A has grown considerably; and when A is zero, as it would be at the beginning of a naive infection, there is no inhibition of virus-infected cell growth. In all the core scenarios presented, we assume that A directly kills C_V, representing the cytotoxic effects of CD8 T cells. This is modeled as a simple mass action interaction between A and C_V occurring at rate k_{va}, to indicate that killing of virus-infected cells by T cells is proportional to the densities of the two interacting populations without saturating effect:

$$-k_{va}A \cdot C_V$$

Altogether we have the following equation for virus-infected cells, C_V, *where in core scenarios we assume* $x_{vainv}=0$, *and only use this when exploring vaccination scenarios*:

$$\frac{dC_V}{dt} = \frac{k_{vg}C_V(1 - k_{vs}C_V)}{1 + (x_{vainv}A)^2} - k_{va}A \cdot C_V$$

Tissue Damage/Dysfunction/DAMPs, *D*, and Innate Immunity/Inflammatory response, *I*

The two interactions for the induction of I by D and the self-recruitment/self-upregulation of I, were modeled with terms that followed the convention used in previous modeling work by (*37*) and (*73*) in which the same two variables were represented but in the context of a generic gram-negative bacterial infection or lipopolysaccharide (LPS) exposure inducing I. The I equation is derived as in (*37*) where a `resting/circulating activated phagocyte' population, call it I_r, is initiated by interaction with pathogen, activated phagocytes, and the presence of damaged tissue. Here the pathogen/virus-infected cells are not included as a direct driver of I. The I_r population is modeled as having a source term, a natural decay term, and an activation term: $\frac{dI_r}{dt} = s_{ir} - \mu_{ir} - R \cdot I_r$, with R as in equation (5). This equation is set to quasi-steady state and the resulting formula for I_r is used in the equation for I which was

originally $\frac{dI}{dt} = R \cdot I_r - \mu_i I$. The function R given by equation (5) consists of terms for the induction of *I* by *D* and the self-recruitment/self-upregulation of I: $k_{id}D + k_{ii}I$. Inhibition of these interactions by the adaptive response, *A*, is also incorporated by dividing by $1 + (x_{iainv}A)^2$ (i.e. multiplying by the function $f_1(1, x_{iainv}A)$ given after equations (1)-(4) above) . Using the above, the following term in equation (3) represents these events:

$$s_{ir}\frac{R}{\mu_{ir} + R} = s_{ir}\frac{\frac{k_{id}D + k_{ii}I}{1 + (x_{iainv}A)^2}}{\mu_{ir} + \frac{k_{id}D + k_{ii}I}{1 + (x_{iainv}A)^2}}$$

In addition, we utilized the same functional form used in (*37*) for both the positive feedback from *I* to *D* and the induction of *D* by C_V. This functional form $f_2(x, y)$ given under equations (1)-(4) above is a hill function with a coefficient of 6 behaves as a steep sigmoidal function, allowing for the increase of *D* to require a threshold-like amount of C_V (and like-wise of *I*) in order for C_V or *I* to induce a substantial amount of *D*, respectively . Thus, low levels of C_V or *I* should not typically induce copious damage/dysfunction. The feedback loop between *D* and *I* may result in mild/recoverable levels of damaged/dysfunctional tissue as a natural process of the host immune response but could escalate in severity, leading to organ failure. Thus, in the equation (2) for *D*, these two interactions are modeled with the terms:

$$k_{dv}f_2(C_V, x_{dv}) + k_{di}f_2(I, x_{di}) = \frac{C_V{}^6}{x_{dv}^6 + C_V{}^6} + \frac{I^6}{x_{di}^6 + I^6}$$

Equation (2) for *D* and equation (3) for *I* have natural decay terms representing respectively repair of damaged tissue $-\mu_d D$ and decay of inflammatory mediators, $-\mu_i I$. Altogether for *D* and *I* we have the following equations:

$$\frac{dD}{dt} = k_{dv}f(C_V, x_{dv}) + k_{di}f(I, x_{di}) - \mu_d D$$
$$\frac{dI}{dt} = s_{ir}\frac{R}{\mu_{ir} + R} - \mu_i I\ .$$

Adaptive Immune Response, *A*

During a viral infection, the initial innate immune response will induce an adaptive immune response for a more specific and effective response against the virus-infected cells. As mentioned earlier, the variable *A* representing the adaptive response serves to directly kill virus-infected cells (i.e. cytotoxic capability of CD8 T cells); and inhibit the infection of other cells (via antibodies). The adaptive response is also responsible for signaling that inhibits the inflammation of the innate immune response as the more specific response is developed. However, if the innate inflammatory response becomes/is quite strong, it can serve to inhibit the initiation/recruitment of the adaptive

response. The adaptive equation also encompasses the role of antigen presenting cells as the component of the response that provides the initiation of the adaptive response by the innate.

The priming of the adaptive response by innate inflammation assumes that a naive *A* population, A_0, is primed by *I* initially but, as *I* becomes larger, it inhibits this induction of *A*:

$$k_{ai}A_0 I \frac{x_{ai}^2}{x_{ai}^2+I^2} .$$

The A_0 population is modeled as $\frac{dA_0}{dt} = S_{a0} - \mu_0 A_0 - k_{ai}A_0 I$, which includes a source term, a natural death term, and a priming term by *I*. This equation is then set to quasi-steady state so that the formula for A_0 becomes $A_0 = \frac{S_{a0}}{\mu_{a0}+k_{ai}I}$ and is inserted in place of A_0, giving the term:

$$k_{ai} \cdot \frac{S_{a0} I}{\mu_{a0}+k_{ai}I} \cdot \frac{x_{ai}^2}{x_{ai}^2+I^2}.$$

The adaptive response includes CD8 T cells that after being primed will undergo clonal expansion. We include a term for this in equation (4) of *A* which assumes that expansion is dependent on the C_V population to indicate immune signaling from virus-infected cells and sensing by the T cells. Also, we assume that expansion of *A* will have a self-saturating effect to account for the self-regulation of the response. The functional form for saturation uses a hill function with hill coefficient 2 to emphasize a steeper threshold-like response to self-regulation:

$$k_{ag} \cdot C_V \cdot \frac{A^2}{x_{ag}^2 + A^2} .$$

As with the other variables, *A*, has a natural decay term, $-\mu_a A$. Also, a source term, A_e, is included to explore an existing source of virus-specific memory cells if considering prior exposure to re-infection or a level of immunity built up over time with a vaccine. Thus, *A* is modeled as:

$$\frac{dA}{dt} = k_{ai} \cdot \frac{S_{a0}}{\mu_{a0} + k_{ai}I} \cdot I \cdot \frac{x_{ai}^2}{x_{ai}^2 + I^2} + k_{ag} \cdot C_V \cdot \frac{A^2}{x_{ag}^2 + A^2} - \mu_a A + A_e .$$

Other Modeling Notes and Considerations

We are not considering a direct negative effect from innate immune factors on the virus-infected cell population. Instead the presence of virus-infected cells will induce signals via *D* that will initiate innate immune mediators, *I*, which in turn initiate specific adaptive immune mediators that can directly kill the virus-infected cells.

Model Parameter Descriptions

Parameter Name	Description	Units	Baseline values & Ranges	Sources and Notes
***C_V*: Virus-infected cell population parameters**				
k_{vg}	Growth/expansion rate of C_V	per day	0.5	Estimated
k_{vs}	$\frac{1}{k_{vs}}$ gives the carrying capacity of C_V	per 10^6 C_V-cells	0.0025	(*33*) (scaled to 10^6 C_V-cells; i.e. Carrying capacity of C_V = 400 x 10^6)
k_{va}	Rate of killing of C_V cells by the adaptive response, *A*	1/*A* per day	0.2	Estimated; (*92*)
x_{vainv}	Controls the strength of *A* to inhibit the growth/expansion of C_V; only utilized for vaccination protocol	per *A*-units	0.0<br>1.0 in vaccination simulations only	Used only in Vaccination and Existing Immunity Simulations
***D*: Tissue Damage/Dysfunction parameters**				
k_{dv}	Rate of production of *D* by C_V	*D*-units/day	0.2	Estimated
x_{dv}	Half-saturation constant of induction of *D* by C_V	10^6 C_V-cells	1.0	Estimated
k_{di}	Maximal rate of damage/DAMPs, *D*, produced by inflammation, *I*	*D*-units/day	0.3<br>0.5 (Moderate)<br>0.8 (Severe)<br>1.0 (Sustained Infl.)	Estimated; (*37*)
x_{di}	Half-saturation constant of induction of *D* by inflammation, *I*	*I*-units	1<br>0.4 (Moderate; Severe; Sustained Infl.)	Estimated
μ_d	Decay rate of *D* (i.e. tissue repair)	per day	0.1	Estimated; (*37*)
***I*: Innate-driven inflammatory response parameters**				
k_{ii}	Maximal rate of self-activation of inflammatory mediators, I_r, by activated innate/inflammatory cells and by-products, *I*	1/*I*-units per day	0.1<br>Set to zero for anti-I therapy simulations	Estimated; (*37*)

s_{ir}	Source of resting/circulating innate/inflammatory cells, I_r	I_r-units/day	0.4	Estimated; (*37*)
μ_{ir}	Decay rate of resting/circulating innate/inflammatory cells, I_r	per day	0.1	Estimated; (*37*)
k_{id}	Rate of activation of resting/circulating innate/inflammatory cells, I_r, by *D*	1/*D*-units per day	0.35<br>Set to zero for anti-I therapy simulations	Estimated; (*37*)
x_{iainv}	Controls the strength of *A* to inhibit the inflammatory response, *I*	per *A*-units	0.1<br>1.0 (Long-lasting Disease)<br>0.5 (Recurrence)	Estimated; (*37*)<br>$\left(\frac{1}{c_\infty}\right)$
μ_i	Decay rate of activated inflammatory cells and by-products, *I*	per day	1.0	Estimated; (*37*)
***A*: Adaptive Immune Response (CD8 T cells/Antibodies) parameters**				
k_{ai}	Rate that innate/inflammatory mediators, *I*, prime naive adaptive T cells (via antigen presenting cells), A_0, into virus-specific CD8 T cells, *A*,	per day	3.0<br>0.1 (Uncontrolled Infection)<br>1.0 (Long-lasting Disease)	Estimated; (*91*)
S_{ao}	Source of unprimed adaptive mediators (naïve T cells)	A-cells/day	1.0	Estimated; (*91, 92*)
μ_{a0}	Decay rate of unprimed adaptive mediators (naïve T cells)	per day	1.0	Estimated; (*91*)
x_{ai}	Half-saturation parameter for inhibition of the priming of *A* by *I*	*I*-units	1.0	Estimated
k_{ag}	Expansion rate of *A* population (i.e. clonal expansion of CD8 T cells and antibody producing cells)	$1/10^6$ C_V -units per day	0.001	Estimated; (*92*)
x_{ag}	Half-saturation parameter for the expansion of *A*	*A*-units	1.0	(*92*)
μ_a	Decay rate of *A*	per day	0.05	Estimated; (*93*)
A_e	Source of existing virus-specific adaptive components/antibodies, *A*	*A*-units/day	0<br>0.05 in Existing Immunity simulations only	Used only for Existing Immunity Simulations
Initial Conditions				
$C_V(0) = 8 \times 10^5$<br>note: $C_V = 10^{-6}$ ($\times 10^6$ V-cells) = 1 cell		$D(0) = 0$	$I(0) = 0$	$A(0) = 0$<br>For $A_e \neq 0$,<br>$A(0) = \frac{A_e}{\mu_a}$

Table S1: Model parameter descriptions, values, and notes. As specified above, the values given are those used to simulate the seven core scenario using the ODE system (1)-(4).

With a parameter set specified, including initial conditions for the variables, the equations are solved with established numerical algorithms for estimating solutions of ordinary differential equations. These algorithms are standard in computational mathematics software such as MATLAB and XPPAUT, both of which were utilized in the analysis and simulation of the model. Parameter values are admittedly difficult to estimate for a model such as this, given the lumped nature of the variables and parameters and the lack of data available for SARS-CoV-2. Many of the model parameters govern the rates of various processes represented in the model (e.g., growth rates, decay rates, activation/priming rates) while others govern the strength of inhibition of one variable on another (e.g., x_{vainv}, x_{iainv}). Still, other parameters determine the levels of variables at which their effects on processes are at half-saturation (e.g., x_{dv}, x_{dv}, x_{ia}, x_{ag}). In any of these roles, the value of any one parameter is essentially tied to an individual's genetic variation, co-morbidities or even lifestyle history.

A baseline set of parameter choices were based on previous work and known inflammatory dynamics (*37*) as well as estimates. The differences between archetypes were due to changes in only four parameters (k_{di}; x_{di}; x_{iainv}; and k_{ai}), while the others were fixed. However, the advantage of modeling is the ability to explore the parameter space to determine the myriad of model behaviors both transiently and in the long-term. Thus, many parameter sets can be explored around the baseline set of parameters. Time courses of the model variables can be generated for each parameter set and together form an interpretable picture of the pathogenesis of the immuno-inflammatory response to the infection, showing both the transient evolution of the variables as well as an overall outcome. Multiple parameter sets can lead to similar manifestations of a response that fall under one of the four major archetypes presented in this manuscript. We chose seven parameter sets that provided a representation of the possibilities and which all arose from changes made to one parameter set such that the overall differences between the number of parameter values differing from one scenario to the next is on the order of three. For instance, the pathways in the model that lead from the Mild recovery scenario to the Moderate recovery scenario to the Severe recovery scenario are all with respect to a change in two parameters (namely the parameters responsible for inducing D via I: k_{di} and x_{di}); though, we acknowledge this is not the only pathway to achieve this progression. Below we describe these parameter changes among the different scenarios relative to the baseline parameter set used for the

Mild Scenario, where these particular modifications do not represent unique ways to achieve these various scenarios/archetypes.

***Recovery* archetype/Moderate scenario (Figure S2), *Recovery* archetype/Severe scenario (Figure 4), and *Sustained Inflammation* archetype (Figure 9)**. The parameter k_{di} is the maximal rate of damage/DAMPs, D, produced by inflammation, I and the parameter x_{di} is the half-saturation constant of induction of D by inflammation, I, in the term, $k_{di}\frac{I^6}{{x_{di}}^6+I^6}$, in the I-equation. The value of x_{di} determines the amount of I needed to reduce k_{di} by half with values of x_{di} smaller than the bounds of I enabling this multiplier term on k_{di} to be an increasing function of I: as I increases, the multiplier on k_{di} is closer to 1. Thus, in the Moderate, Severe, and Sustained Inflammation scenarios, the effect of *increasing* k_{di} (from 0.3 in Mild to 0.5 in Moderate; or 0.8 in Severe; or 1.0 in Sustained Inflammation) and *decreasing* x_{di} (from 1.0 in Mild to 0.4 in Moderate, Severe, and Sustained Inflammation) is to make the production of D by I progressively more pronounced compared to Mild leading to a stronger D to I feedback and thus more sever disease progression.

Recurrence Scenario – *Recrudescence* Archetype (Figure 6). The parameter x_{iainv} controls the strength of A to inhibit the inflammatory response, I. Thus, an increase in this parameter will increase the inhibitory effect on I by A. In the Recurrence scenario (of the *Recrudescence* archetype), this parameter was *increased* from the value of x_{iainv}=0.1 in the Mild Scenario to a value of x_{iainv}=0.5 and, in addition, the parameter x_{di}=1.0 in Mild was *decreased* to 0.5 (See above discussion on this parameter).

Long-lasting Disease Scenario – *Recrudescence* Archetype (Figure 7). As described above for the Recurrence scenario, an increase to the x_{iainv} parameter will increase the inhibitory effect on I by A. The parameter k_{ai} is the rate that innate/inflammatory mediators, I, prime naive adaptive T cells (via antigen presenting cells), A_0, into virus-specific CD8 T cells, A. For the Long-lasting Disease scenario (of the *Recrudescence* archetype), the parameter x_{iainv} was *increased* from the value of 0.1 in the Mild Scenario to a value of x_{iainv}=1.0; and the parameter k_{ai} was *decreased* from a value of 3.0 in the Mild Scenario to k_{ai} =0.1. This had the combined effect of weakening the feedback mechanisms between A and I which lead to stunting the ability of I to prime A enough to suppress C_V completely, leading to a situation in which virus-infected cells are not eliminated yet remain at lower, perhaps undetectable levels that continue to drive a lengthy immune response having signs of recovery only to be followed

by reprise. This gave rise to the scenario name has it reflected descriptions by some health professionals of patients who struggle with the disease progression/symptoms for months at a time.

***Uncontrolled Infection* Archetype (Figure 8)**. As in the Long-lasting Disease scenario, the Uncontrolled Infection scenario decreased the parameter k_{ai} from a value of 3.0 in the Mild Scenario to k_{ai} =0.1. However, all other parameters were the same as that for the Mild case. This gave rise to a more severe instance of an inadequate immune response (relative to that in the Long-lasting Disease scenario) which causes uncontrolled growth of the virus-infected cell population as well as sustained inflammation.

Parameters involved in treatment Simulations. Simulations of corticosteroid therapy modified parameters k_{ii} and k_{id} to have values of zero during the therapy window as described in the text. These two parameters are rates of induction of inflammation, *I*, respectively by the innate inflammatory response itself through self-enhancement and by damage-induced mechanisms. Thus, setting these two parameters to a value of zero during a window of time essentially shuts off any production of I completely for that window. After the treatment window ends, these two parameters are restored to their pre-treatment values.

Vaccination Protocol Implementations: short-term vaccine effects and existing immunity.

We implemented two different vaccination-type scenarios in the core scenarios. One, termed 'short-term vaccination,' looks at the short-term effects of a vaccine that is adjuvant-based and where a COVID infection happens withing 2 weeks of the last dose. The other, termed "existing immunity," looks at considering already established, existing level of virus-specific immunity at the time of infection.

For the short-term vaccination, a function $J(t) = e^{-k_{adj}t} = e^{-3t}$ was defined to represent an exponentially decaying stimulus of the *I* response and was incorporated within the function *R* in the *I* equation to emulate induction of *I* by an adjuvant which decays over time. The induction of *I* leads to the priming of *A*, the specific adaptive response to the virus. If C_V is present, the *A* response will be magnified in a C_V-dependent manner as described in model development. The decay period of the adjuvant stimulus function was chosen with initial condition at Day 0 normalized to a value of 1.0 and exhibiting exponential decay at a rate such that the stimulus is negligible by Day 3.

The parameter, x_{vainv}, governs the strength of A to inhibit the growth/expansion of the C_V population. This parameter is set to 0 when not implementing a vaccination protocol, thus only allowing for direct (e.g., cell-mediated) killing of C_V in the model without vaccination. However, for vaccination we assume $x_{vainv} > 0$ (specifically, $x_{vainv} = 1.0$) to allow the vaccine to be responsible for the induction of antibodies needed to prevent virus from infecting more cells, and therefore inhibiting the expansion of the virus-infected cell population, C_V, in addition to the direct killing of C_V by A. There is no build-up mechanism/equation of long-term memory yet modeled and thus the vaccination effects are assumed to be viable for the short-term, which drove the decision to initiate an infection 14 days after the second vaccine dose was simulated initiated (with $8x10_5$ virus-infected cells). The two vaccine doses administered 28 days apart was chosen to emulate current vaccine protocols (*94*).

To simulate an established build-up of immunity, we utilized the parameter A_e in the A equation to represent an existing source of A and set this parameter to a nonzero amount ($A_e = 0.05$) and used $x_{vainv} = 1.0$ both chosen so that the effects would not be unrealistically too weak or too strong (data not shown) in inhibiting C_V. Thus, at time zero when an infection is initiated (with $8x10_5$ virus-infected cells), A levels would not be zero (as in the other scenario) but instead $A(0) = \frac{\mu_A}{A_e}$ and therefore able to respond immediately to the viral threat.

	Mild	Hosp	Long Hosp/ICU	Sustained Inflammation	Uncontrolled Infection	Long-lasting Disease	Recrudescence
$C_V(0)$							
1	35.55	35.55	35.5	35.5	46.25	43.55	35.85
10	30.95	30.95	30.9	30.9	41.65	38.95	31.25
100	26.35	26.3	26.3	26.3	37.05	34.35	26.60
1000	21.75	21.70	21.70	21.70	32.45	29.75	22
10^4	17.15	17.1	17.1	17.1	27.85	25.15	17.40
10^5	12.55	12.5	12.5	12.5	23.25	20.55	12.80
**8x10^5**	**8.50**	**8.50**	**8.45**	**8.45**	**19.05**	**16.30**	**8.75**
10^6	8.25	8.20	8.20	8.20	18.65	15.75	8.50

Table S2: Exploration of the impact of increasing initial viral load on the time to peak infection. The days to peak C_V level as a function the initial C_V were calculated for each of the seven core modeling scenarios and for eight different initial doses of C_V ranging from the equivalent of one virus-infected cell to one million virus-infected cells. Time to virus positivity was calculated from incidental COVID-19 patients as 11.3±5.3 (n=8; range 4-22) days as discussed in the main text. From these data, we calibrated to a C_V peak time of 11.1±4.5 days (range 8.5-19.1 days) in subsequent simulations based on an initial C_V dose equivalent to 8x10^5 virus-infected cells (bolded row).

Archetype	Labels for scenarios within archetype	Description
Recovery	Mild	Recovery at home may be feasible or brief hospitalization required
	Moderate	Hospitalization required with or without ICU
	Severe	
Sustained Inflammation	Sustained Inflammation	Hospitalization or ICU required; Longer than average stays; Non-recovery
Uncontrolled Infection	Uncontrolled Infection	Hospitalization or ICU required; Longer than average stays; Non-recovery
Recrudescence/ Persistent Disease	Long-lasting Disease	Moderate or severe persistent symptoms and disease-associated side effects experienced without or with hospitalization over a longer time period than hospitalization scenarios in the Recovery archetype; recrudescence may be the cause even if under threshold of detection; Final outcome ambiguous
	Recrudescence	Mild to Severe disease course and symptoms due to a reprisal of viral-infected cells potentially giving a positive PCR after a previously positive, then negative PCR result; Final outcome ambiguous

Table S3: Labels and descriptions of scenarios within archetypes used in the manuscript.
